# Advanced Computational Biology Methods Identify Molecular Switches for Malignancy in an EGF Mouse Model of Liver Cancer

**DOI:** 10.1371/journal.pone.0017738

**Published:** 2011-03-28

**Authors:** Philip Stegmaier, Nico Voss, Tatiana Meier, Alexander Kel, Edgar Wingender, Juergen Borlak

**Affiliations:** 1 BIOBASE GmbH, Wolfenbuettel, Germany; 2 Department Molecular Medicine and Medical Biotechnology, Fraunhofer Institute of Toxicology and Experimental Medicine, Hannover, Germany; 3 Centre for Pharmacology and Toxicology, Hannover Medical School, Hannover, Germany; 4 GeneXplain GmbH, Wolfenbuettel, Germany; 5 Institute of Chemical Biology and Fundamental Medicine, Novosibirsk, Russia; 6 Department of Bioinformatics, University of Goettingen, Goettingen, Germany; Texas A&M University, United States of America

## Abstract

The molecular causes by which the epidermal growth factor receptor tyrosine kinase induces malignant transformation are largely unknown. To better understand EGFs' transforming capacity whole genome scans were applied to a transgenic mouse model of liver cancer and subjected to advanced methods of computational analysis to construct de novo gene regulatory networks based on a combination of sequence analysis and entrained graph-topological algorithms. Here we identified transcription factors, processes, key nodes and molecules to connect as yet unknown interacting partners at the level of protein-DNA interaction. Many of those could be confirmed by electromobility band shift assay at recognition sites of gene specific promoters and by western blotting of nuclear proteins. A novel cellular regulatory circuitry could therefore be proposed that connects cell cycle regulated genes with components of the EGF signaling pathway. Promoter analysis of differentially expressed genes suggested the majority of regulated transcription factors to display specificity to either the pre-tumor or the tumor state. Subsequent search for signal transduction key nodes upstream of the identified transcription factors and their targets suggested the insulin-like growth factor pathway to render the tumor cells independent of EGF receptor activity. Notably, expression of IGF2 in addition to many components of this pathway was highly upregulated in tumors. Together, we propose a switch in autocrine signaling to foster tumor growth that was initially triggered by EGF and demonstrate the knowledge gain form promoter analysis combined with upstream key node identification.

## Introduction

Epidermal growth factor is an important mitogen for hepatocytes for its ability to modulate proto-oncogene as well as liver specific gene expression. To better understand EGF's role in malignant transformation a transgenic mouse model was developed where EGF was targeted to the liver. Notably, transgenic mice developed liver cancer around 7–8 months and a tumour stage-dependent network of EGF-regulated genes was identified, as previously reported [1]. Encouraged by these findings genes linked to tumorigenes and progression of disease could be proposed. Here, we wished to analyze gene expression profiles of pre-tumorous and highly differentiated hepatocellular carcinomas with a novel computational method that enabled identification of regulators of the EGF signalling cascade associated with malignant transformation. A new method was developed based on promoter sequence analysis of differentially expressed genes. Specifically, transcription of a gene is determined to a major part by the activity of transcription factors, which in turn recognize specific short DNA segments, i.e. transcription factor binding sites (TFBSs) which are often situated in the promoter region upstream of the transcription start site (TSS). Gene expression profiles can thus be used to identify TFs that potentially influence the expression of genes under certain cellular conditions by use of various genetic algorithms and matrices that recognise TFBSs. The complexity of the gene expression data can then be reduced by identification of common TFs of co-regulated genes. The here described and newly developed method focuses on the identification of transcription factor binding sites with co-occupancy in the promoters of differentially expressed genes in a statistically significant manner. This enabled hypotheses generation and an identification of transcription factors acting on such a promoter set with the ultimate goal to identify “molecular triggers” in gene regulatory networks forcing hepatocytes into malignant transformation. Based on such analysis transcription factors were identified as candidate effectors of malignant transformation which may function in the switch from EGF over expression to the malignant state. In order to experimentally validate the computational predictions Western blotting experiments of nuclear proteins and EMSA band shift assays were carried out to determine the DNA binding activity of several transcription factors. Reconstruction of signalling cascades upstream of these TFs allowed us to suggest the downstream targets of EGF signalling in these two types of cellular states, i.e. transgenicity and liver cancer. As a result, we propose regulatory networks that help to better understand EGF-induced malignancies. In an effort to search for key molecules in the signalling network upstream of the identified transcription factors the insulin-like growth factor pathway was identified that indeed may represent a molecular switch from the EGF receptor tyrosine kinase route to the tumour state thereby rendering malignantly transformed cells independent of EGF receptor activity. Further evidence for this hypothesis was obtained when the gene expression of IGF2 and its down stream partners was investigated and determined to be highly significantly induced in tumour cells as were many components of this pathway.

Overall this study aims for a better understanding of the EGF transforming capacity and combines different lines of evidence for a possible mechanism of disease.

## Results

### Differential expression in transgenic (pre-tumor) and tumor tissue: sub classification of known liver cancer biomarkers by gene expression profiles

**Figure 1:**
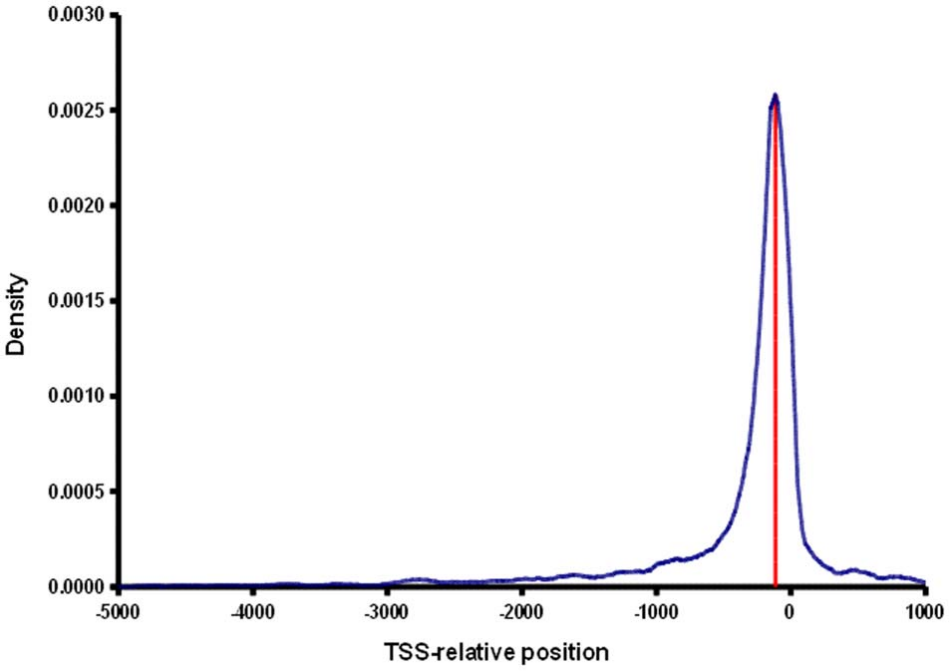
Distribution of known transcription factor binding site locations (blue) according to the TRANSFAC database release 12.1. The red line indicates the peak of the distribution at –115bp relative to the TSS.

ExPlain 2.3 mapped Affymetrix probe sets to 10,262 mouse genes. Differential expression analysis with Ebarrays detected 303 and 355 up regulated genes as well as 95 and 141 down regulated genes in transgenic and tumor cells, respectively. Table 1 summarizes information about obtained gene lists and Figure 1 depicts the distribution of known transcription factor binding site locations according to the TRANSFAC database release 12.1 (see Material and Methods, calculation of P-values for MATCH scores, for further details). Table S1 provides all MGI gene symbols, TRANSPATH molecule names, if available, and highest or lowest fold changes measured for probe sets of up regulated or down regulated genes, respectively. A minor manual modification was introduced to gene set 5 (Table 1), where a probe set mapped to four closely related paralogs Bcl2a1a–d. We used the MAFFT alignment software [2] and Jalview [3] to inspect the multiple alignments of Bcl2a1 promoters (Figure S1). Since promoter sequences of these genes are very similar, a bias in promoter analyses performed in this work could be expected and we therefore removed three Bcl2a genes (b–d) from gene set 5.

**Table 1:** Sets of differentially expressed genes in transgenic and tumor cells defined by statistical analysis and fold changes.

Gene set	Progression state	Fold change in other condition	#genes
**Upregulation**			
1	**Transgenic**	Fold change ≤2 in tumor cells	82
2		Fold change >2 in tumor cells	77
3	**Transgenic & Tumor**		144
4	**Tumor**	Fold change >2 in transgenic cells	103
5		Fold change ≤2 in transgenic cells	108
**Downregulation**			
6	**Transgenic**	Fold change ≥0.5 in tumor cells	47
7		Fold change <0.5 in tumor cells	23
8	**Transgenic & Tumor**		25
9	**Tumor**	Fold change <0.5 in transgenic cells	24
10		Fold change ≥0.5 in transgenic cells	92

Transgenic and tumor states shared 144 up regulated genes and 25 down regulated genes (sets 3 and 8, Table 1). In subsequent analyses we also considered a potentially larger overlap, when a probe set was statistically significant in one contrast and achieved a high fold change in the other (sets 2, 4, 7, and 9). Thus, for 77 of the 303 up regulated genes in transgenic cells (set 2), a probe set detected by statistical analysis also had a fold change >2 in tumor cells, and these 77 genes were added to the 355 genes up regulated in tumor to obtain an extended set of up regulated genes in tumor cells. Correspondingly, 103 of the 355 genes up regulated in tumor were appended to the transgenic set to derive an extended set of up regulated genes in transgenic cells (set 4). Likewise, gene sets of the down regulation response were enlarged at a fold change below 0.5. Remaining subsets (sets 1, 5, 6, and 10) were considered specifically regulated in the respective progression state.

**Figure 2:**
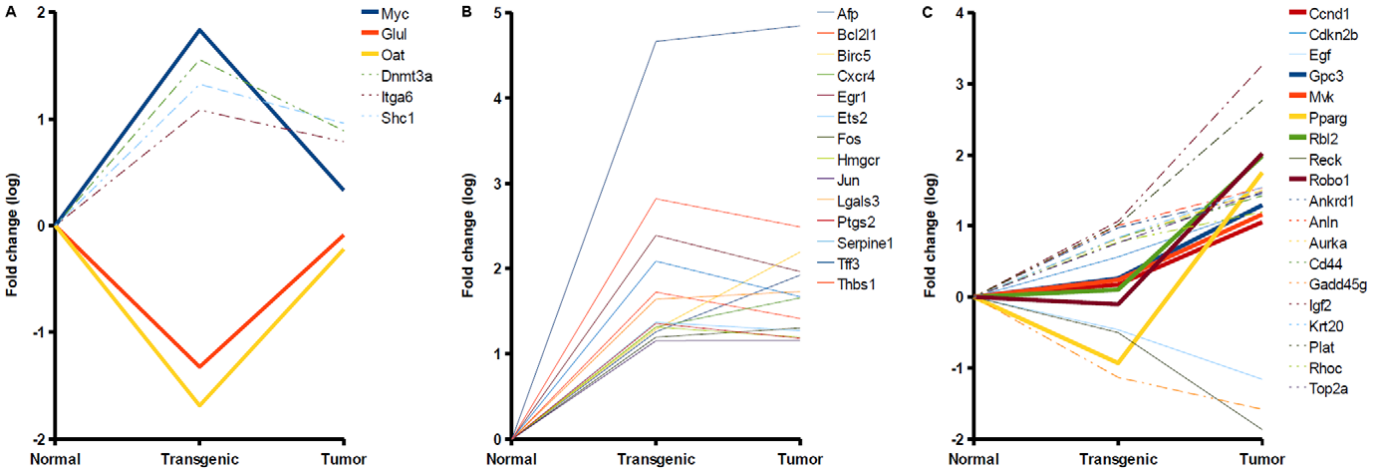
Expression responses of known liver carcinoma/neoplasia biomarkers in EGF-induced carcinogenicity. The plots show log-Fold changes of known biomarkers which were differentially expressed in transgenic cells (A), both states (B), or tumors (C). Fold changes of some genes indicated differential expression in both states, although statistical analysis assigned them to one state only (dashed lines). Several known biomarkers exhibited progression state-specific responses, which may subserve derivation of pre-tumor and tumor signatures (bold lines).

According to the disease module of the BIOBASE Knowledge Library (BKL) [4], EGF-induced carcinogenesis caused differential expression of 39 known biomarker genes associated with liver carcinoma/neoplasms (Table S1). As shown in Figure 2, these biomarkers featured different patterns of expression suggesting a further sub classification with regard to their response in pre-tumor and tumor state. Three genes, namely Myc, Glul, Oat, were transiently up- or down regulated during disease onset and may thus serve as early markers for liver cancer, which discriminate against the tumor state (Figure 2A). Statistical analysis also suggested Dnmt3a, Itga6, and Shc1, however high fold changes were measured for these genes in tumor cells as discussed above. Expression of 14 biomarker genes changed detectably in both progression states (Figure 2B) indicating an additional set of putative early liver cancer markers. Finally, Ccnd1, Gpc3, Mvk, Pparg, Rbl2, and Robo1 exhibited a tumor-specific response (Figure 2C), where adverse expression changes were observed for Pparg, which appeared down regulated in transgenic cells (fold change <0.4) and significantly up regulated in tumors. Taken together, these results suggest a refined interpretation of known biomarkers for liver cancer/neoplasms. Among respective genes we could identify several informative signatures that indicate specific pre-tumor and tumor markers and show that expression changes of some known biomarkers may in fact serve as early indicators of disease onset.

### Regulation of cell cycle and lipid metabolism changes progressively in EGF-induced hepatocarcinogenesis

**Figure 3:**
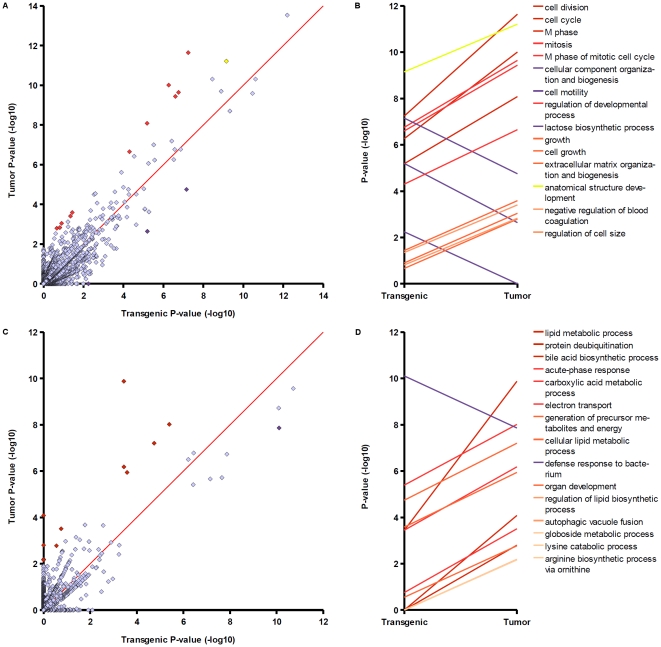
Comparison of Gene Ontology Biological Process enrichment in transgenic and tumor gene sets. (A and B – analysis of upregulated genes, C and D – analysis of downregulated genes). A and C) Each dot represents a GO category in the space of two P-values. B and D) Top 15 GO terms with greatest log-P-value difference between transgenic and tumor. Dots corresponding to categories listed in B and D are highlighted in A and C, respectively.

With defined sets of differentially expressed (DE) genes at hand, we set out to identify functional changes that accompany EGF-induced hepatocarcinogenesis. For this purpose, we calculated enrichment P-values for all GO Biological Process categories associated with at least one gene in transgenic or tumour gene sets and applied them as a measure of relative importance of a particular biological function for a given gene set. To limit the effect of false negative findings during differential expression analysis, enrichment P-values were calculated for extended gene sets composed of sets 1–4 (up regulated in transgenic cells), 2–5 (up regulated in tumor cells), 6–9 (down regulated in transgenic cells), and 7–10 (down regulated in tumour cells) (Table 1). Results of the P-value comparison are shown in Figure 3. In the following we focus on the 15 GO groups with largest differences between log-P-values as obtained in analyses of either upregulated or downregulated gene sets. Plots of transgenic versus tumour P-values (Figure 3, A and C) illustrate that the procedure ordered categories according to their distance to the diagonal (red line). Points on the diagonal indicate no difference between P-values. In the selected top 15 biological processes, P-values varied by about 2–6 orders of magnitude between transgenic and tumour states. According to this analysis, upregulation of genes during tumorigenes most strongly altered cell cycle functions (Figure 3B). Note that legends of Figures 3B and 3D preserve the ordering by log-P-value difference. All of the top five GO categories, “cell division”, “cell cycle”, “M phase”, “mitosis”, and “M phase of mitotic cell cycle” allude to changes in cell cycle and were more significantly enriched in the tumor gene set, whereas upregulation in transgenic cells was more strongly directed at mechanisms of cell motility as well as cellular component organization and biogenesis. Besides alterations of cell cycle, functional comparison points out changes in regulation of genes taking part in developmental processes, cell growth, and anatomical structure development (Figure 3B), which imply potential dedifferentiation events. Analysis of downregulation responses reveals that regulation of lipid metabolism was strongly modified during tumorigenesis. The next two highest ranked functions pertain to protein deubiquitination and bile acid synthesis (Figure 3D). Table S2 provides additional details about genes matching some selected biological process groups and their P-values in either progression state. Cell cycle categories highly ranked by comparison of upregulated gene sets were significantly affected in both transgenic and tumor cells, which shows that these functions undergo progressive alterations. In particular, genes associated with anatomical structure development were strongly enriched in transgenic and tumor sets (yellow dot and line, Figure 3, A and B). Notably, differences manifested not only in upregulation of additional genes in the tumor cell; e.g. the cell cycle group of transgenic cells comprises Foxc1, Gadd45a, Hic1, Hus1, Myc, and Uhmk1, which were not detected in tumor (despite extension of the gene list). The most dramatic changes were observed in regulation of lipid metabolic genes. Transgenic and tumor gene sets involved in this function differed by 21 genes and enrichment P-values increased by about six orders of magnitude. Furthermore, protein deubiquitination and bile acid metabolism exhibit a switch-like regulation, where differential expression was first detected in the tumor.

Cell-cycle dysregulation was previously identified as one causal mechanism of nongenotoxic carcinogenicity [5]. It is also known that liver cancer entails lipid metabolic derangements including cholesterol metabolism [6]. The results presented here show that disease onset was accompanied by progressive changes in respective functions. The ubiquitin-proteasome pathway is a relatively new target for cancer therapy [7]. According to our gene expression data, hepatocarcinogenesis caused downregulation of three deubiquitination genes, Dub1, Dub2, and Dub2a, specifically in the tumor state (Table S1). Recently, a deubiquitinating enzyme, BAP1, with a role in cell cycle regulation was described as tumor suppressor [8], supporting the relevance of finding the corresponding GO category among the top 15 altered functions despite a moderate enrichment P-value in the tumor gene set (P<0.0001). Downregulation of deubiquitination genes complies with previous findings, as Ventii and coauthors also observed deficiency in deubiquitinating activity in cancer-associated mutants. In summary, progressive changes in regulation of cell cycle, developmental and lipid metabolic functions chaperoned EGF-induced hepatocarcinogenesis, whereas potential switch-like regulation was observed for small groups of genes defined by protein deubiquitination and bile acid biosynthesis, a component of hepatic cholesterol metabolism. These biological functions may harbor novel biomarkers for disease onset and tumorigenesis.

### Clusters of upregulated signal transduction molecules in transgenic and tumor cells integrate growth factor, cell cycle, chemokine and cytokine signals

**Figure 4:**
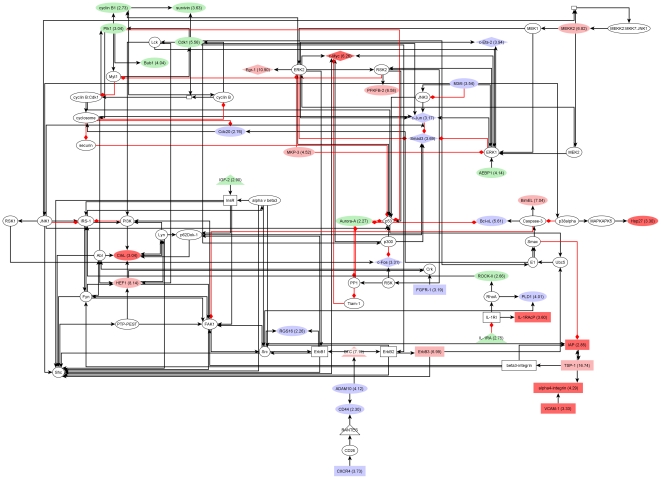
Network of upregulated signaling components in transgenic cells. Red nodes: only in transgenic network; Light red: fold change was at least +2 higher than in tumor; Light green: fold change was at least - 2 lower than in tumor, Blue: upregulated molecule with similar fold change in transgenic and tumor. Please, see text for further description.

**Figure 5:**
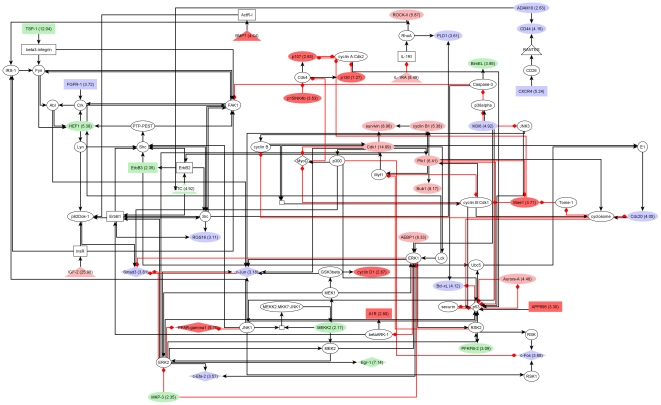
Network of upregulated signaling components in tumor cells. Red nodes: only in tumor network; Light red: fold change was at least 2 points higher than in transgenic cells; Light green: fold change was at least 2 points lower than in transgenic cells, Blue: upregulated molecule with similar fold change in transgenic and tumor. Please, see text for further description.

Networks of interacting proteins exert a large part of cellular functions. Analysis of differentially expressed molecules in the context of known signalling pathways enables identification of molecular networks targeted by observed expression changes. We applied network cluster analysis to propose functional context for signaling components encoded by differentially expressed genes of transgenic and tumor cells along with supporting network topologies constructed from experimentally proven signaling reactions. Networks were constructed for extended upregulation and downregulation gene sets described above. As a result, we obtained one cluster each for upregulated genes of transgenic cells and for upregulated genes of tumor cells. A small network of downregulated genes was found in tumor cells, in which EGF and beta-c interact with Shc and a complex comprising EGF, ErbB1, Shc-1, Grb2, and Sos (not shown). The two networks of upregulated genes were constituted by 85 and 88 components including 39 and 41 molecules upregulated in transgenic or tumor cells, respectively. Diagrams of transgenic and tumor network clusters are shown in Figures 4 and 5. Further information about differentially expressed molecules and encoding genes is given in Table S3.

Both networks feature a mix of functional categories such as growth factor signaling, e.g. through ErbB receptors, InsR, or FGFR-1, cell cycle-regulatory cascades, e.g. involving cyclin B1, Cdk1, Aurora-A, or p53, as well as cytokine and chemokine signaling through IL-1RI and CXCR4. The combined list of upregulated components consists of 48 proteins of which 7 are specific to the transgenic network and 9 are only part of the tumor network (Table S3). For 19 of the 32 shared molecules a quantitative change in expression during tumorigenesis may be hypothesized as indicated by node coloring on network diagrams (green and light red nodes, Figures 4 and 5). Notably, this applies to a compact module of cell cycle regulators, namely survivin, cyclin B1, Cdk1, Plk1, and Bub1, whose expression increased about 2-fold in tumor versus transgenic cells according to measured fold changes. Moreover, network analysis suggests another, tumor-specific module of cell cycle regulators formed by p107, p130, p15INK4b, and Wee1 (Figure 5). These results further support the hypothesis that EGF-induced carcinogenesis is driven in part by progressive alterations of cell cycle regulation, which, as the networks show, may also manifest through quantitative changes of expression.

The presented network clusters reveal potential effects on the activity of upregulated TFs like c-Fos, c-Jun, c-Myc, PPAR-gamma1, Smad3, Egr-1, and c-Ets-2. C-Myc and PPAR-gamma1 were restricted to the transgenic and the tumor network, respectively. Each factor integrates signals from several upstream molecules with altered expression. Interestingly, well-known cancer-associated TFs like c-Fos, c-Jun, Smad3, and c-Ets-2 exhibited rather constant levels of upregulation throughout tumorigenesis. Furthermore, a relatively high number of both activating and inhibitory reactions target p53, which was void of detectable differential expression in either progression state.

A cascade involving VCAM-1, alpha4-integrin, and IAP was found specifically in the transgenic network (Figure 4). Alpha4-integrin shows progression state-specific regulation with upregulation in transgenic cells and downregulation in tumor cells (Table S3). Integrins are linked to tissue invasion by hepatocarcinoma cells [9] and play a role in apoptosis [10]. BMP7, a tumor network-specific molecule, can act as transcription factor and may as such contribute to upregulation of c-Myb [11] as well as of c-Fos, the latter possibly by other means [12]. BMP7 was previously reported to participate in regulation of apoptosis in vascular smooth muscle cells [13]. Given their association with apoptosis and their progression state-specific expression profiles, alpha4-integrin and BMP7 may represent constituents of switch mechanisms of carcinogenesis.

The network clusters reveal regulatory circuitries that might be explored for novel therapeutic interventions. Indeed, PPAR-gamma antagonists are being investigated as treatment of various malignancies including liver cancers. Regulatory cascades targeting PPAR-gamma through upstream kinases and phosphatases, such as M3/6, JNK1, MEKK2, MKP3, MEK2, or ERK2, of which M3/6, MEKK2, and MFP-3 were induced during carcinogenesis, suggest additional possibilities for drug development. Furthermore, the ligand of insulin and insulin-like receptors, IGF-2, was strongly upregulated in tumor cells, whereas there were moderate changes in transgenic cells (not detected by differential expression analysis). The potential role of this ligand in autocrine regulation of cancer cell growth was previously discussed in the literature [14] and further analyzed in our study (see below).

### Promoter analysis and identification of regulatory sequences

Transcription factors are important contributors to coordinated gene expression changes like those observed in the study data. It is a standard approach to test for overrepresentation of TF binding sites in promoters of coregulated genes versus a background of promoters. We quantified binding site enrichment by the 0.01-quantile of the ratio of two Beta distributions modeling the odds ratio of predicted binding sites and promoters and foreground and background gene sets. The 0.01-quantile value, in the following denoted *q-value*, estimates the value of the odds ratio, so that the true ratio is higher with 99% probability (see Methods). For each of the 578 TRANSFAC PWMs, the algorithm started with a low PWM score threshold (P-value 0.05) and iteratively adjusted the cut-off (by incrementing) to maximize the q-value.

Binding sites were predicted by MATCH in promoter regions covering −1000 to +100 relative to the TSS. We constructed separate background sets for transgenic and tumor cells by randomly sampling 1000 genes with fold changes between 0.9 and 1.1 in the respective progression state. The following foreground sets were analyzed: upregulation (Table 1: sets 1–3 and 3–5), specific upregulation (Table 1: sets 1 and 5), downregulation (Table 1: sets 6–8 and 8–10), and specific downregulation (Table 1: sets 6 and 10). In addition, binding site enrichment was tested in promoters of upregulated genes associated with cell cycle and of downregulated genes associated with lipid metabolism.

**Figure 6:**
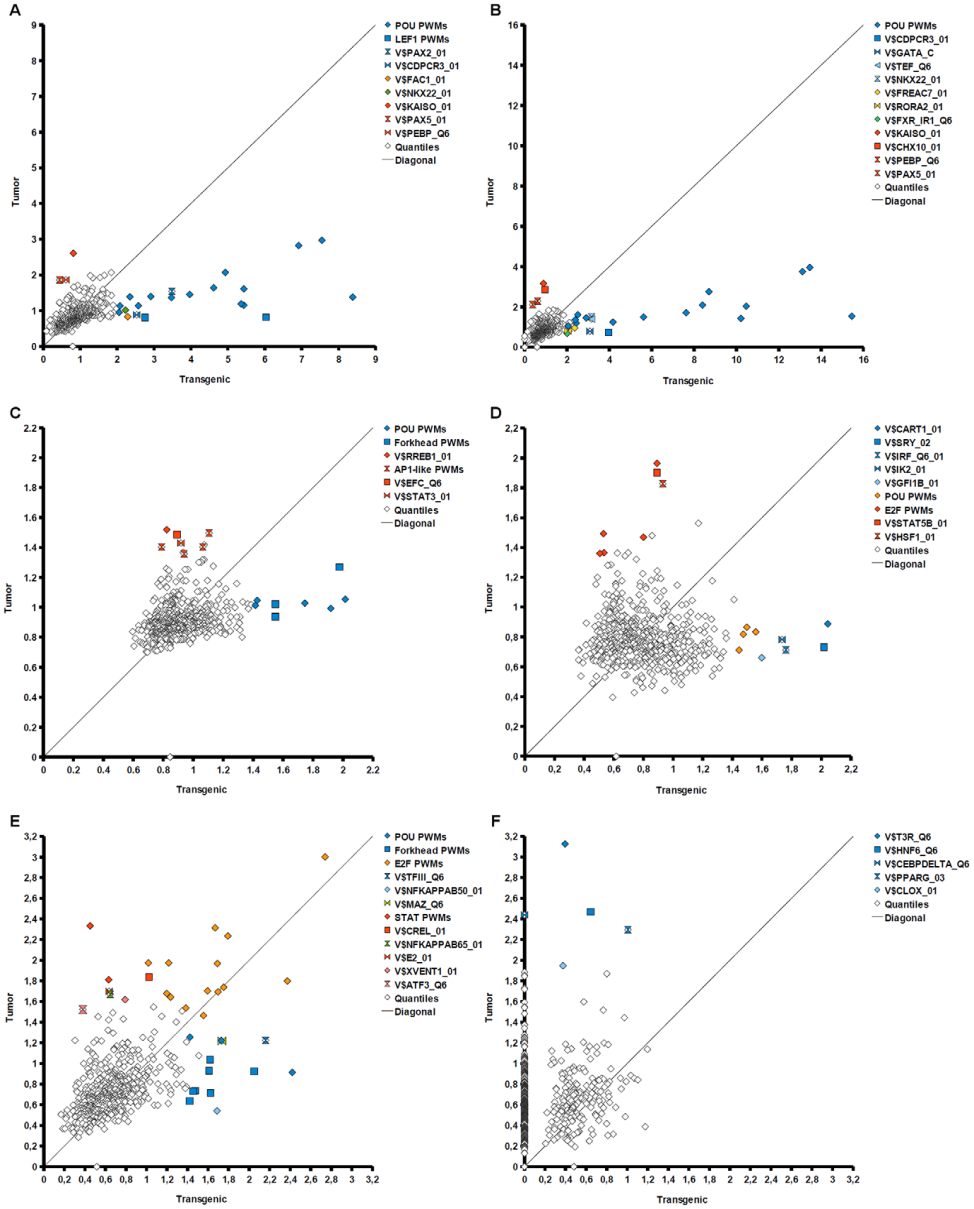
Enrichment of TF binding sites in transgenic and tumor DE gene sets. Each dot represents one TRANSFAC binding site ( = sequence motif) positioned by fold enrichment in transgenic (x-axis) and tumor (y-axis) foreground sets. Quantile values greater than 1 indicate binding site enrichment. Several motifs are highlighted which were shifted away from the diagonal suggesting different importance of corresponding TFs for regulation in either transgenic or tumor states. Please, see text for further description. A) Analysis of binding site enrichment in all down regulated genes B) Analysis of binding site enrichments in specifically down regulated genes of either transgenic or tumor states C) Analysis of binding site enrichment in all up regulated genes D) Analysis of binding site enrichments in specifically up regulated genes of either transgenic or tumor states E) Analysis of binding site enrichments in up regulated cell cycle genes F) Analysis of binding site enrichments in down regulated lipid metabolism genes.

In Figure 6, q-values of TRANSFAC motifs optimized for transgenic foreground sets are plotted against q-values of corresponding tumor foreground sets. Furthermore, we extracted the top PWMs ordered by q-values in Table S4. Identifiers of TRANSFAC matrices whose dots are highlighted in Figure 6 are bold-typed in Table S4. Extraction of matrices followed the rule to show the top 15 PWMs, or all with at least 2-fold enrichment in either transgenic or tumor set, or all PWMs highlighted in Figure 6, whichever resulted in the largest number of motifs. We also extracted transcription factor genes (Table S5) according to identified PWMs (underlined in Table S4) and performed upstream network analysis with transcription factor sets (see below).

As a result, promoter analysis revealed TF motifs specifically, or more articulately overrepresented in transgenic or tumor foreground sets as well as motifs with common enrichment in both progression states. In 5 of 6 foreground sets, POU motifs were more strongly associated with the transgenic state than with the tumor state (Figure 6A–E). This difference was most pronounced in analyses of downregulated (Figure 6A) and specific downregulated gene sets (Figure 6B), where dots representing POU matrices (blue diamonds) are located far away from the mass of points. Notably, sites of some POU motifs were also more than 2-fold enriched in promoters of downregulated genes in tumor. Oct1 matrices were the top ranked motifs in downregulated and specific downregulated genes in tumor (Table S4). However, promoters of upregulated genes were detectably enriched with POU sites in the transgenic state only (Figure 6C–E). These results suggest that POU factors contributed to the switch from transgenic to tumor state. Furthermore, their activity may represent a major cause of observed downregulation events. Among the POU transcription factors represented by matrices identified in the analyses, the expression profile of Pou5f1 (Oct4) resembled well the observed progression-state specific enrichment of its binding sites (Table S5). The Pou5f1 gene exhibited a high fold change (2.75) in the transgenic state, which had decreased in the tumor state (fold change 1.70). Indeed, the Pou5f1 gene is specifically expressed in embryonic stem cells and in tumor cells, but not in cells of differentiated tissues [15]. Transcription factors with a Forkhead domain also showed association with the transgenic state. This signal was best observed in upregulation and cell cycle gene sets (Figure 6Cand E), yet subtle enrichment in transgenic promoters could also be detected in specific downregulation (Figure 6B) and specific upregulation, where the FREAC2 motif ranked among the top 15 PWMs (Table S4). In the upregulation set, Foxd3 binding sites showed the strongest signal after Oct1 sites (Table S4). This would support a potential role of Oct4, as corepression through overlapping binding sites of Oct4 and Foxd3 was previously reported [16]. According to expression measurements, Foxd3 was potentially downregulated in both progression states (Table S5), although measured expression differences were not statistically significant. Instead, Foxc1 expression parallels the stronger enrichment of Forkhead binding sites in transgenic promoter sets, as it is specifically upregulated in the early progression state (Tables S1 and S5).

Promoters of upregulated genes in tumor were associated with binding sites of cell cycle regulators such as AP1-like factors, STAT, and E2f (Figure 6C–E), of which Atf3, Jun, and E2f3 were significantly upregulated in both transgenic and tumor cells (Tables S1 and S5). This finding supports the stronger regulation of cell cycle processes in tumor detected by comparative GO analysis. The analysis of cell cycle gene promoters suggests E2f factors as the most important regulators in both states, whereas a tendency towards higher q-values in the tumor set was observed for several E2f motifs (Figure 6E). Notably, the Myc-associated zinc finger protein was detected in the transgenic cell cycle gene set (Tables S4 and S5), which indirectly suggests that Myc impacted cell cycle regulation in transgenic cells, but not or to a lesser extent in tumor cells. This would be supported by the expression profile of Myc with significant upregulation in the early state and subtle or absent upregulation in tumor.

Finally, the lipid metabolism gene sets show strong association of HNF6 (Onecut1) and PPAR-gamma with the tumor state (Figure 6F). Of these, HNF6 was significantly downregulated in tumor, whereas PPAR-gamma exhibited a progression state specific profile with downregulation in the transgenic state and significant upregulation in tumor.

While many of the aforementioned transcription factors are well-known proto-oncogenes, such as Jun, Myc, or E2f3, and the link between HNF6, PPARgamma and lipid metabolism is comprehensible, other factors revealed by our analysis are novel with respect to their role in liver carcinogenesis. Binding sites of Kaiso (Zbtb33) were most strongly overrepresented in downregulated tumor genes. Kaiso was shown to silence tumor suppressor genes in colorectal cancer [17], and its role in cancer was previously reviewed [18]. Furthermore, motifs of HMG box factors were associated with transgenic gene sets in downregulation (Figure 6A, LEF1 PWMs) and specific upregulation (Figure 6D, Sry). While the Lef1 gene was moderately upregulated in transgenic and tumor cells (Table S5), Tcf7 showed significant upregulation in tumor. Also, Tcf7l2 appeared to be induced in tumor compared to its expression level in transgenic cells (Table S1). All in all, Tcf7 and Lef1 factors are known to play a role in Wnt signaling, which indicates a connection between these TFs and Kaiso target genes [18]. Moreover, Wnt signaling components and the HMG box factor Sox2 were previously implicated in Oct4-dependent transcriptional networks [19]. Hence, these findings suggest an EGF-induced mechanism of dedifferentiation and establishment of stem cell-like properties, which may have been driven by Oct4 on the level of transcription. The carcinogenetic mechanism may therefore share similarities with embryonic stem cell signaling pathwaysf, which was further supported by enrichment of developmental pathways in Gene Ontology analysis.

### Key node network analysis reveals a key role for IGF-2 signaling in carcinogenesis

**Figure 7:**
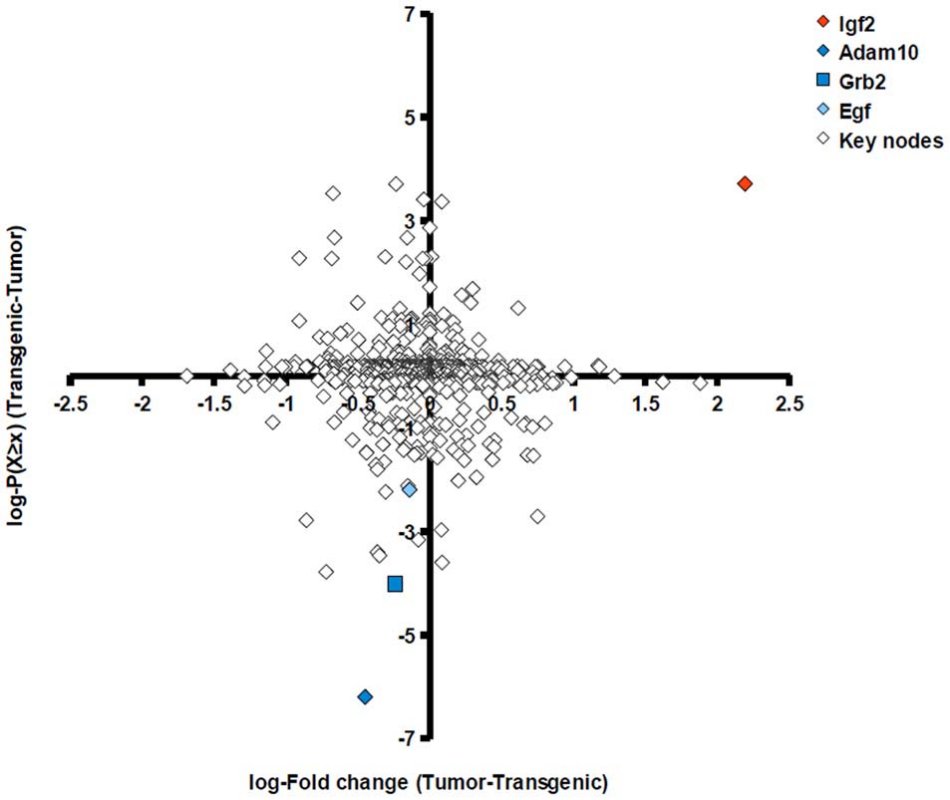
Plot of expression fold changes versus score statistics of key nodes. Each dot represents one key node found upstream of transcription factors identified by promoter analysis. Coordinates are the difference of log-Fold changes (x-axis) and the difference of log-probabilities of key node scores (y-axis). Higher values correspond to higher expression or higher rank of the key node score, respectively, in the tumor state. Components of the EGF pathway were associated with the transgenic state (blue dots), whereas Igf2 is associated with the tumor state with respect to both expression and key node score difference.

Given transcription factors whose binding sites were enriched in transgenic or tumor gene sets, we sought upstream regulators of these TFs that may contribute to their activation or inhibition through signal transduction pathways. We extracted TFs linked to overrepresented TRANSFAC motifs (Table S4, underlined PWM names) and subjected resulting TF sets to key node analysis. Network analyses were carried out separately for transgenic and tumor. Again, we compared transgenic and tumor results. We first calculated the empirical cumulative probability P(X≥x), in the following denoted ECP, for each key node. For this, key nodes reported by ExPlain were ranked by their key node score (see Material and methods). The ECP of a key node was then computed on the basis of its rank among all key nodes. Figure 7 shows a plot of log-ECP differences (tumor-transgenic) against differences of log-Fold change values (tumor-transgenic). Dots representing key nodes with strongest difference in importance for transgenic or tumor TF sets are located far away from the ordinate origin. Fold change differences were depicted on the abscissa.

Notably, components of the EGF signaling network, Adam10, a metallopeptidase that processes EGF, and Grb2, an adaptor protein that binds directly to the EGF receptor, exhibited a greater importance for transgenic than for tumor TFs (Figure 7, blue dots). We also highlighted EGF, which occupied a less extremal position (Figure 7, light blue dot). Here we find IGF-2 as tumor-associated key node, whose location reflects both a greater importance for tumor TFs as well as increased expression in the tumor state (Figure 7, red dot). IGF-2 is a ligand of the insulin receptor as well as the insulin-like growth factor 1-receptor (IGF-1R). Notably, the shift of IGF-1, another IGF1-R ligand, also indicated association with the tumor state, yet the IGF1 gene was potentially downregulated in tumor cells (data not shown).

**Figure 8:**
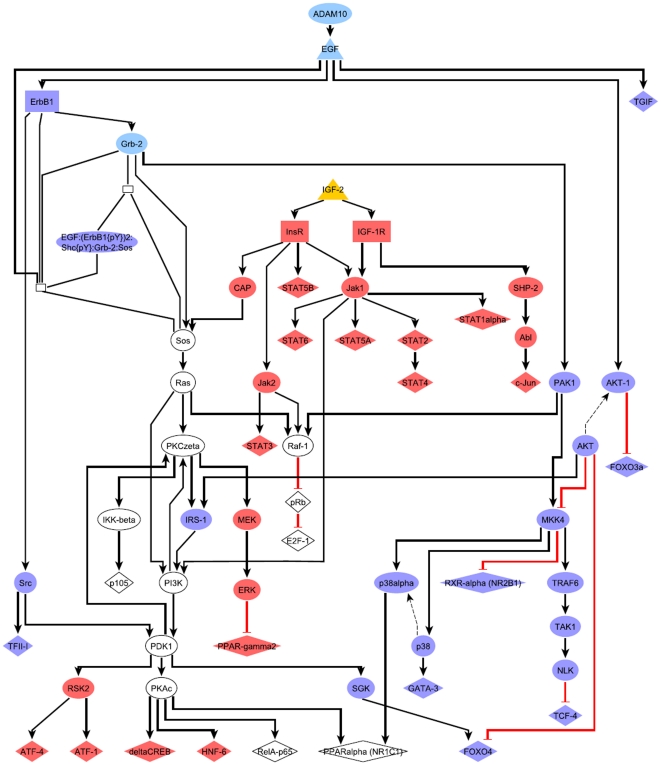
Merged key node networks of EGF and IGF-2 cascades with transcription factors revealed by promoter analysis. Blue nodes: EGF-specific network components, Red: IGF-2-specific network components, Light blue: Key nodes of the EGF pathway, Yellow: IGF-2.

To examine the role of IGF-2 signaling as a surrogate for EGF, we superposed the key node/TF networks of Adam10, Grb2 and EGF with the IGF-2 network (Figure 8). The merged network shows that IGF-2 may at least in part substitute for EGF signaling (white nodes are shared by IGF-2 and EGF pathways). Although in that network AKT cascades are only connected to EGF-specific pathways, IGF-1R is also known to activate AKT [20]. Recalling that EGF was downregulated and IGF-2 was strongly upregulated in tumor cells, the level of IGF-1R dysregulation may represent a switch that marks the onset of malignant transformation. This is further supported by evidence that tumor cells utilize IGF-1R signaling as a survival mechanism that renders them independent of EGF signals [21]. Notably, our findings suggest an interplay between EGF and IGF-2 pathways already at early stages of carcinogenesis.

### Analysis of TF co-occurrences in promoters of upregulated tumor genes

To further investigate the EGF-induced switch from transgenic to tumor state, we performed another step of promoter analysis, where we focused on TFs, whose regulation of activity can be mechanistically explained by signal transduction cascades downstream of an EGF receptor ligand or an EGF receptor. Hence, our goal was to identify EGF-dependent transcription regulators that could exert the switch from the transgenic to the tumor state. Therefore, we sought TFs downstream from EGF whose binding sites were enriched in promoters of upregulated tumor genes. This was done using the key node functionality of ExPlain. Co-occurrence analysis of TF binding sites was chosen to achieve higher specificity in selecting potentially regulated promoter regions in foreground and background sets than is possible by considering individual PWMs only.

We retrieved all TFs that were inferred in the signal transduction network downstream from the EGF ligands and receptors: HB-EGF, EGF, ErbB1, ErbB2, or ErbB3. In addition, we included PWMs of upregulated tumor factors. TRANSFAC matrices linked to all such TFs were used for further analysis. In total, we obtained 266 “tumor-EGF-network” PWMs yielding 31,306 pairwise combinations. To avoid pairs of similar matrices, we considered only combinations of PWMs, which overlapped in less than 10% of the hits of the PWM with the lower total number of matches at a score P-value threshold of 0.001. Score P-values were estimated on the basis of predictions in promoters of the background subset. As a result, 34 and 45 PWM pairs were reported at a P-value below 10^−3^ in the tumor complete and specific gene sets, respectively. Identified pairs grouped by TF classes are provided in Table S6.

**Figure 9:**
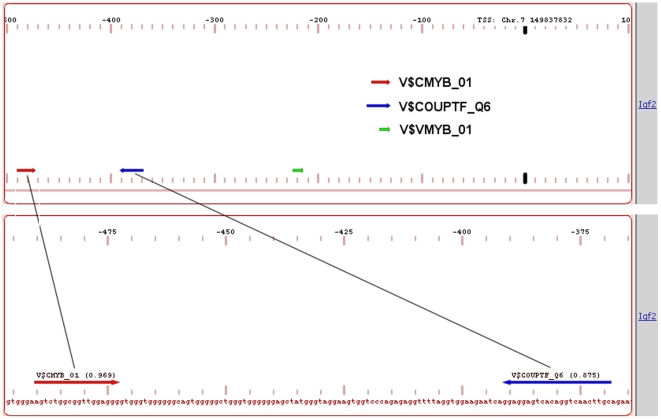
Putative binding sites for c-Myb and HNF4/COUP-TF transcription factors in the promoter of the Igf2 gene. Regulation of Igf2 gene can be explained in part by the binding of c-Myb to its recognition site in the promoter of this gene.

The TF pair analysis results provide evidence for involvement of several upregulated TFs such as Atf3, E2f3, Egr1, Fos, Hnf1b, Jun, Maff, Mef2a, Nfe2, Nfe2l3, Nr2f1 or Nr2f2, Pbx3. Pparg, Smad3, and Tcf7. These factors were found in different combinations in the pair analysis (Table S7). We additionally found overrepresented pairs of MEF with the known protooncogene c-Myb. MEF mediates G1-S mitotic transition, erythrocyte differentiation, and regulation of myeloid cell differentiation. It is known to be upregulated in breast cancer as well as several other neoplasms. C-Myb was previously shown to cooperate with various other transcription factors such as factors from the Ets family but also with C/EBP and AML factors [22]. In our analysis we detected co-occurrence of Myb binding sites and HNF4/COUP bindings sites (Table S6). Notably, the Myb – COUP-TF pair was predicted in the promoter of the Igf2 gene, which was significantly upregulated in the tumor conditions (Figure 9). We think that indeed upregulation of this gene can be explained by a transactivation effect of c-Myb combined with the potential competitive substitution of the tumor downregulated HNF4 factor by COUP-TF1. The COUP-TF1 gene (Nr2f1) was upregulated in transgenic and tumor in comparison to the normal tissue according to gene expression measurements (fold changes 6.26 and 2.62 in transgenic and tumor, respectively). The antagonistic competition of HNF4 with COUP-TF factors for the same promoter element has been described before, e.g. for human ApoCII [23] and for human EPO [24].

### Analysis of multiple combinations of TF binding sites in promoters of upregulated and downregulated genes in tumor

**Figure 10:**
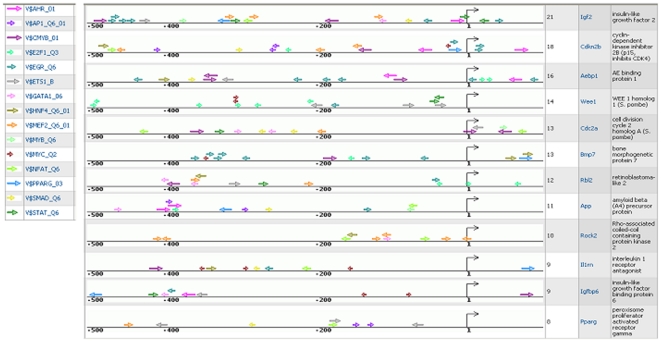
Maps of predicted binding sites of selected TRANSFAC weight matrices in promoters of genes up- or downregulated in tumours versus transgenic cells.

Synergistic binding of multiple transcription factors to certain combinations of binding sites is an important mechanism of achieving highly specific regulation of genes in particular cellular conditions. We can hypothesize, that tumor cells are characterized by particular pattern of activated transcription factors, which enabled them to evolve from pre-tumor to tumor state by escaping multiple checkpoints and by establishing signal independent uncontrolled proliferation. We sought to identify such patterns of multiple activated transcription factors, which synergistically bind to their target genes and may be responsible for the transgenic-tumor switch. For this, we focused our attention, first of all, on genes specifically upregulated in tumor compared to transgenic state, that encode components of the revealed EGF and IGF-2 pathways. Such genes can provide complex feedback mechanisms in the network helping to maintain the tumor status of the cells. All highly upregulated genes (log-Fold change in tumor > 2.0) were mapped on the TRANSPATH network and signal transduction chains propagating the activation signal downstream to transcription factors were constructed using ExPlain tools. Among the components of such downstream signal transduction network we identified 12 proteins whose genes switched expression in the transgenic-tumor transition (transgenic fold change < tumor fold change). We speculate that the change of expression of these 12 genes in tumor compared to transgenic contributes to self-maintenance of the tumor state, and the set of transcription factors regulating activity of these genes might be the best candidates for understanding the regulatory mechanism of this switch. In addition, we analyzed 34 genes, that were downregulated in tumors compared to transgenic state and that were associated with the most significantly downregulated functional category “organ development” as revealed by GSEA analysis. We analyzed promoters of these upregulated and downregulated genes using the ExPlain tool in order to reconstruct maps of multiple TF binding sites in promoters of these genes, which could help us to understand the molecular mechanisms of the switch to the tumor state. TF site maps of upregulated gene promoters are depicted in Figure 10. It is obvious that most of the promoters are characterized by similar composition of TF binding sites, although, their mutual location and position in the promoters relative to the start of transcription can vary significantly between different promoters.

### Western blotting and DNA binding activity of networked transcription factors at gene specific promoters

**Figure 11:**
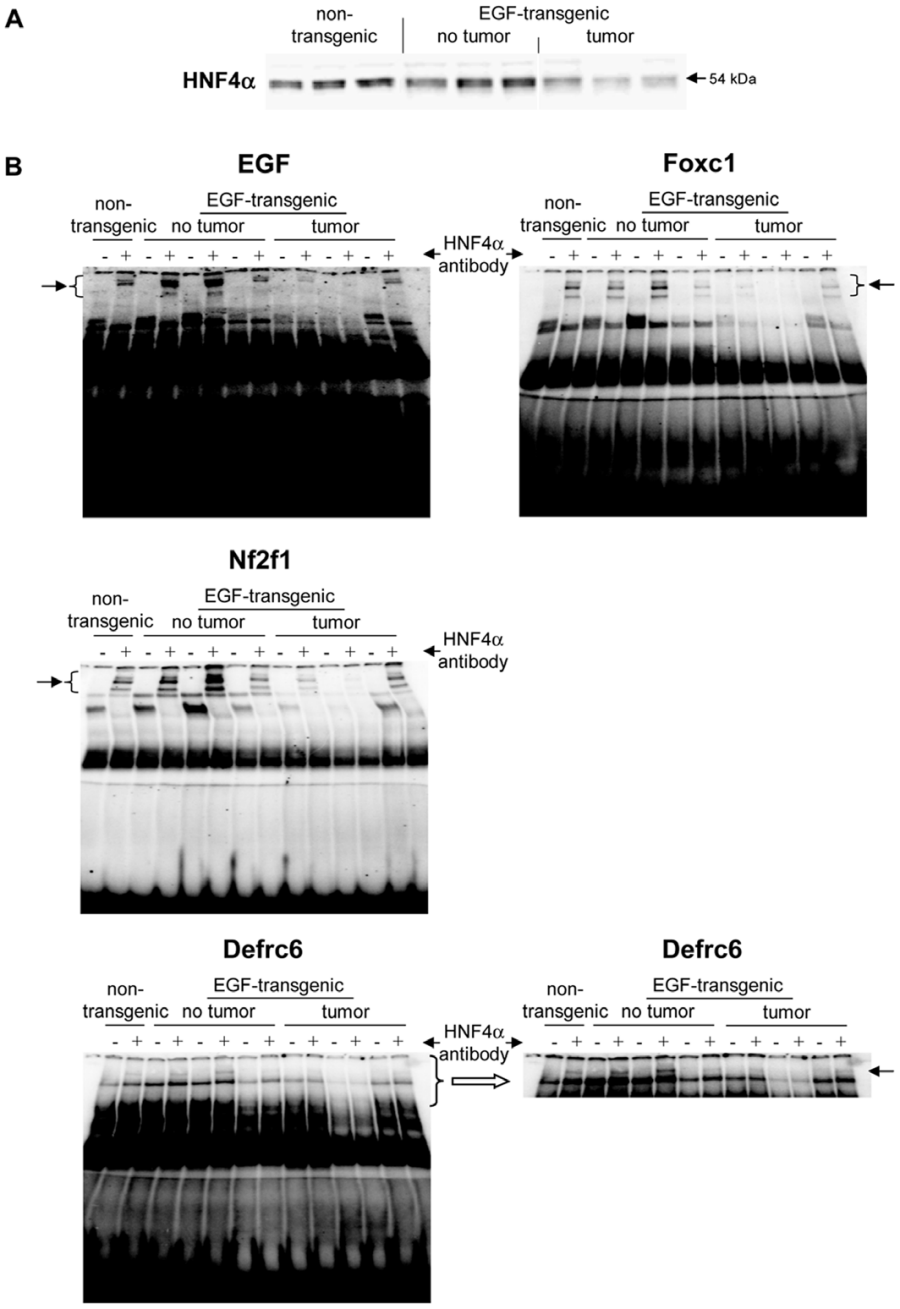
Western blotting and EMSA for HNF4α. **A** Western blotting of HNF4α with nuclear protein extracts isolated from liver tissue of non-transgenic, transgenic and tumour tissue **B** EMSA confirmation experiments at gene specific promoter sites of EGF, Foxc1, Nf2f1 and Defrc1.

**Figure 12:**
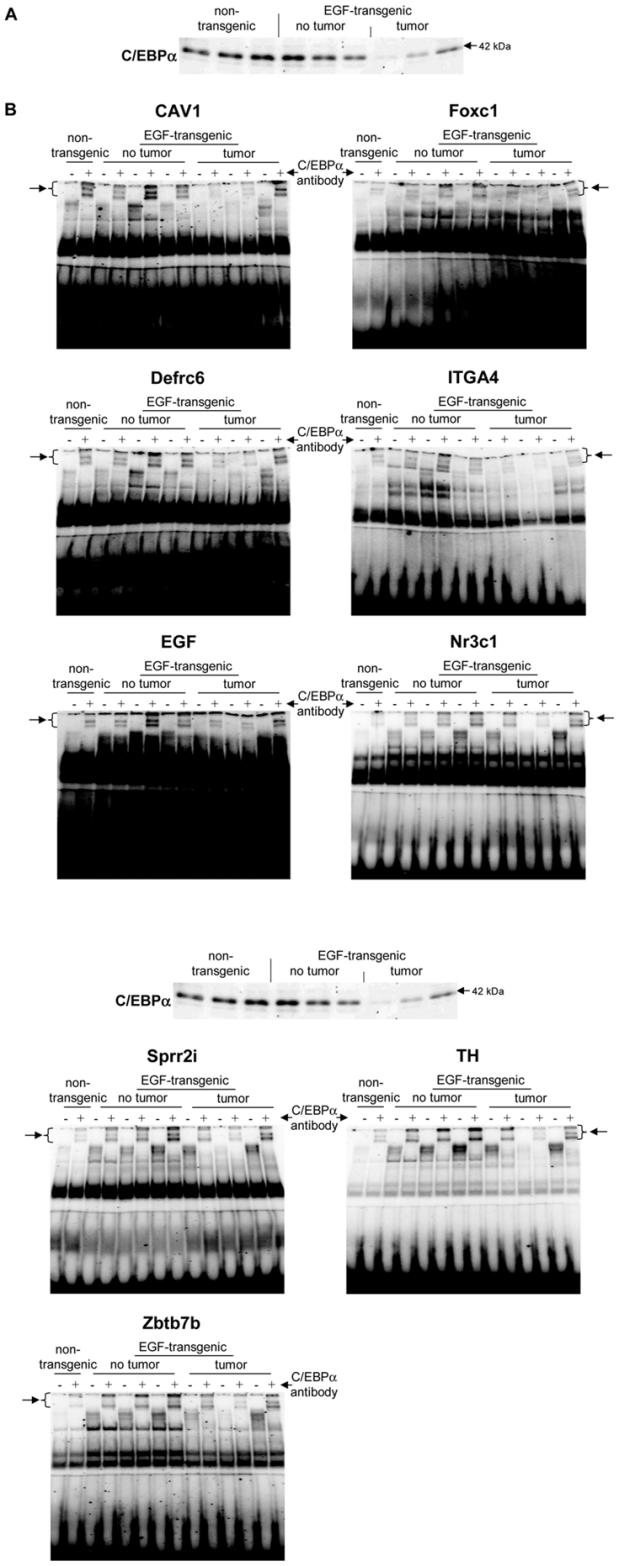
Western blotting and EMSA for CEBPα. **A** Western blotting of CEBPα with nuclear protein extracts isolated from liver tissue of non-transgenic, transgenic and tumour tissue **B** EMSA confirmation experiments at gene specific promoter sites of CAV1, Foxc1, Defrc1, ITGA4, EGF, Nr3c1, Sprr2i, TH and Zbtb7b.

**Figure 13:**
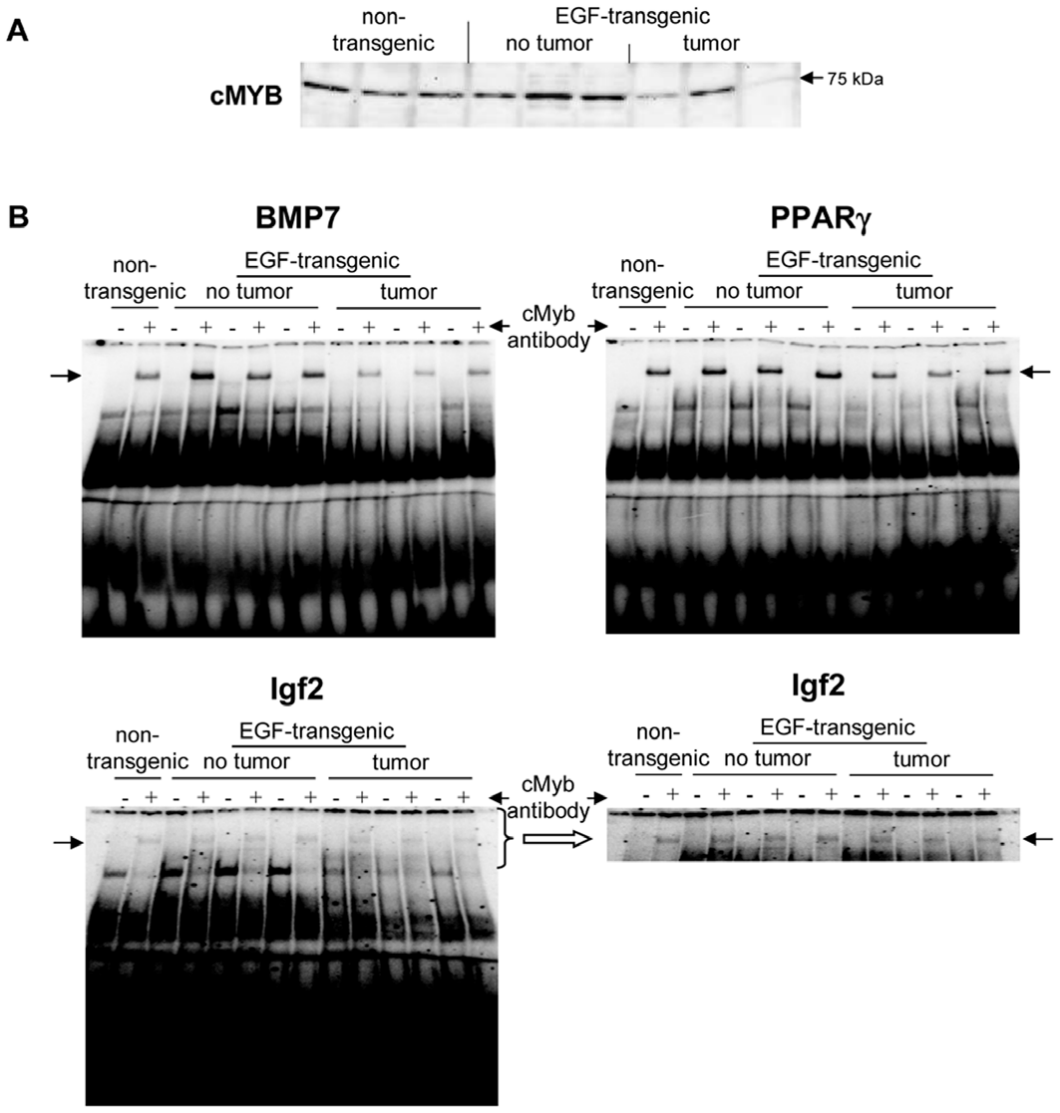
Western blotting and EMSA for cMYB. **A** Western blotting of cMYB with nuclear protein extracts isolated from liver tissue of non-transgenic, transgenic and tumour tissue **B** EMSA confirmation experiments at gene specific promoter sites of BMP7, PPARγ and IGF2.

**Figure 14:**
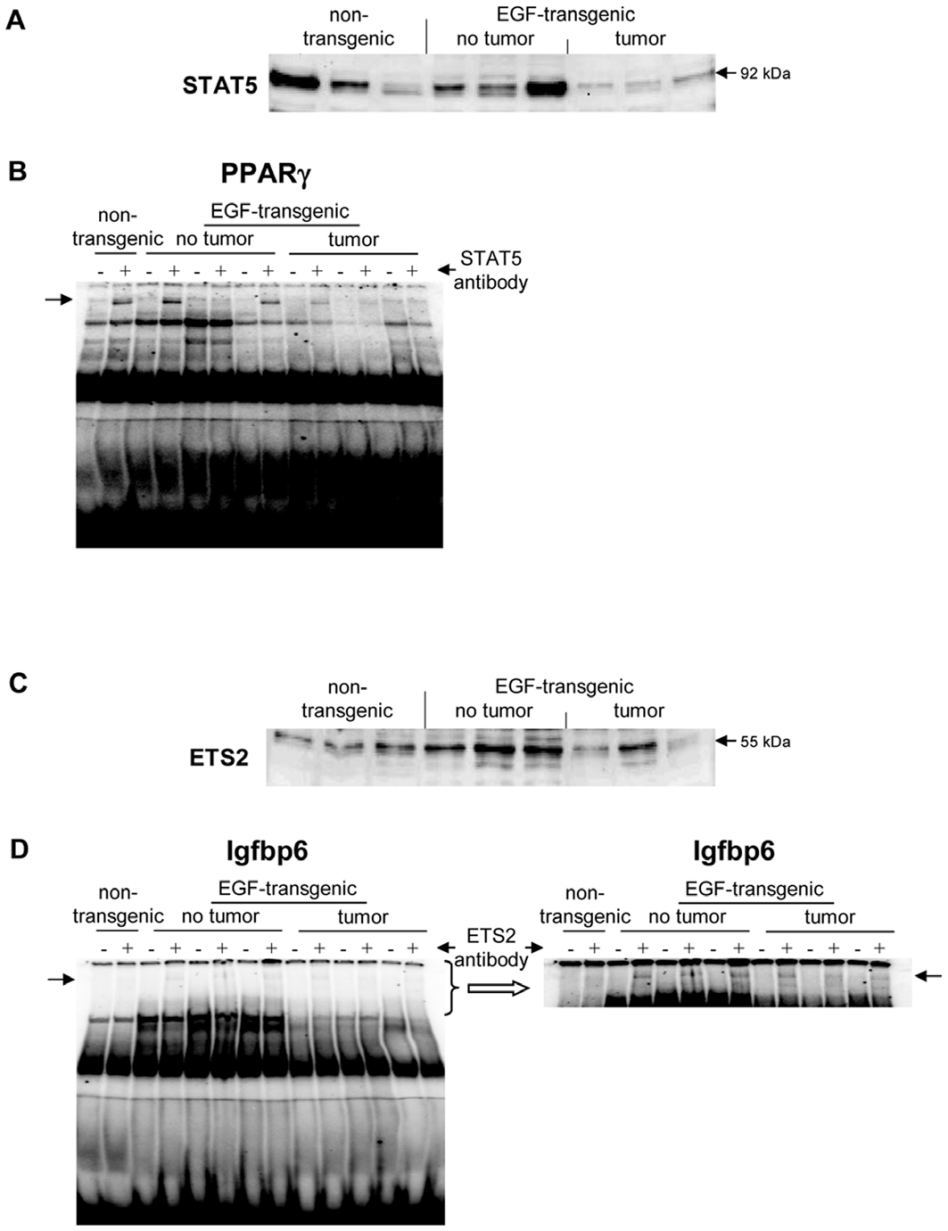
Western blotting and EMSA for STAT5 and ETS2. **A** Western blotting of STAT5 with nuclear protein extracts isolated from liver tissue of non-transgenic, transgenic and tumour tissue **B** EMSA confirmation experiments at gene specific promoter sites of PPARγ **C** Western blotting of ETS2 with nuclear protein extracts isolated from liver tissue of non-transgenic, transgenic and tumour tissue **D** EMSA confirmation experiments at gene specific promoter sites of Igfbp6.

**Figure 15:**
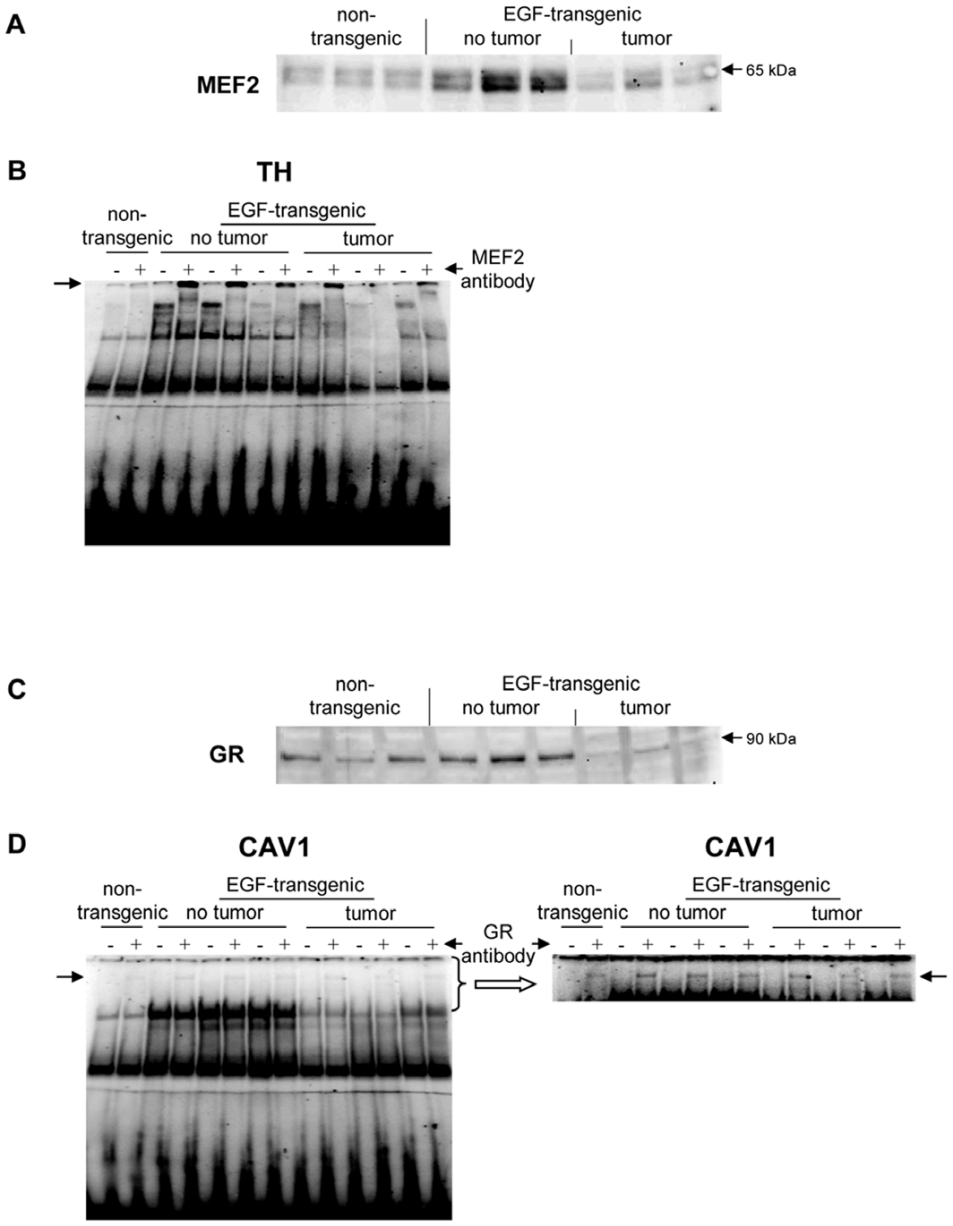
Western blotting and EMSA for MEF2 and GR. **A** Western blotting of MEF2 with nuclear protein extracts isolated from liver tissue of non-transgenic, transgenic and tumour tissue **B** EMSA confirmation experiments at gene specific promoter sites of TH **C** Western blotting of GR with nuclear protein extracts isolated from liver tissue of non-transgenic, transgenic and tumour tissue **D** EMSA confirmation experiments at gene specific promoter sites of CAV1.

**Figure 16:**
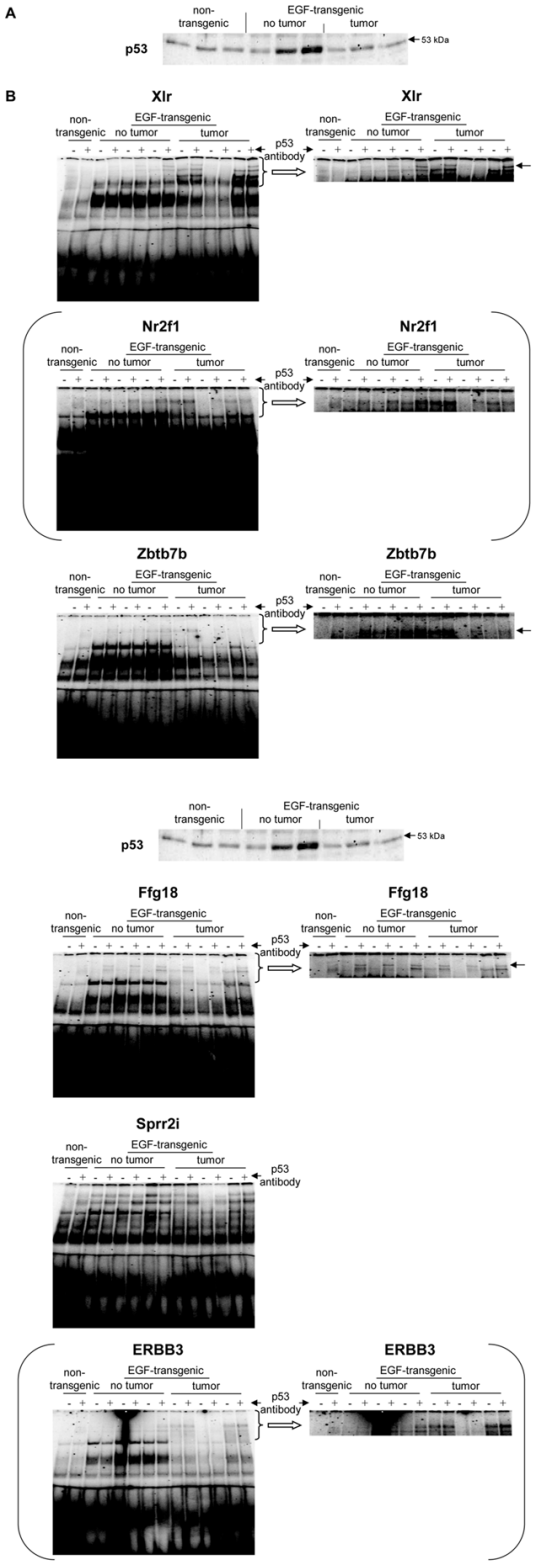
Western blotting and EMSA for p53. **A** Western blotting of p53 with nuclear protein extracts isolated from liver tissue of non-transgenic, transgenic and tumour tissue **B** EMSA confirmation experiments at gene specific promoter sites of Xlr, Nr2f1, Zbtb.7b, Ffg18, Sprr2i and ERBB3.

**Figure 17:**
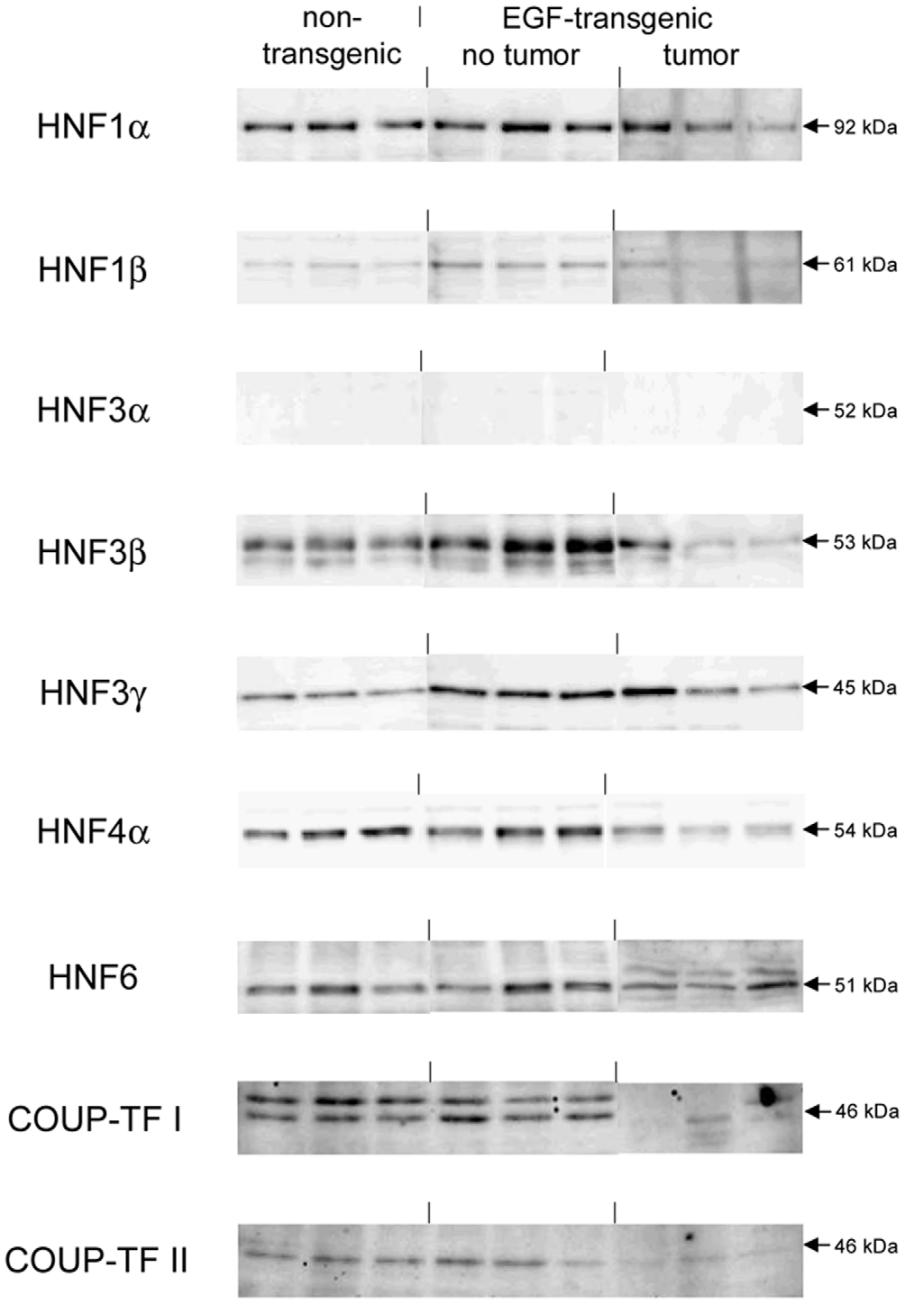
Western blotting of liver enriched transcription factors with nuclear protein extracts isolated from liver tissue of non-transgenic, transgenic and tumour tissue.

A total of 5 upregulated and 14 downregulated genes were selected based on highest scores for predicted TF binding sites while EMSA band shift assays were carried out with specific antibodies to fully validate the predicted DNA binding activity. As shown in Figure 11A Western blotting experiments with nuclear extracts of tumor tissue revealed highly significant repression of HNF4alpha, while DNA binding activity of this Zn-finger protein was evidenced at gene specific promoters of EGF, Foxc1, Nfsf1and Defrc6. Here, the DNA binding activity varied, when individual promoters were analyzed but recapitulate the findings from the Western blotting experiments, i.e. the HNF4alpha binding activity followed the order > transgenic without tumor > non-transgenic and tumor extracts, respectively. Likewise, expression of the CAAT enhancer binding protein C/EBPalpha was particularly repressed with nuclear extracts of tumor tissue (see Figure 12A) while EMSA band shift (Figure 12B) assays confirmed its binding at gene specific promoters of Cav1, Foxc1, Defrc6, Itga4, EGF, Nr3c1,Sprr2i, TH and Zbtb7b, albeit at different levels. Notably, little to none DNA binding activity was observed with nuclear extracts of tumor tissue. Furthermore, the DNA binding activity of c-Myb was studied. As shown in Figure 13A its expression was significantly reduced in nuclear extracts of tumor tissue of two of the three individual animals investigated, but its DNA binding activity appeared to be particularly reduced at gene specific promoter sequences of BMP7 but less so at predicted TF binding sites of PPARg and Igf2. Very significant regulation was also observed for STAT5 with almost none detectable expression levels in nuclear extracts of tumor tissue even though one non transgenic control animal also displayed very low levels of this transcription factor (Figure 14A). Consequently, its DNA binding activity at gene specific promoter sequences of the transcription factor PPARg was nearly non detectable (Figure 14B). As observed with other transcription factors (see above) expression of ETS was repressed in nuclear extracts of tumor tissues (see Figure 14C) while its DNA binding activity was hardly measurable at predicted TF binding sites at gene specific promoter sequences of Igfbp6 (Figure 14D). The expression and DNA binding activity of the transcription factor Mef2 was also investigated (Figure 15A). Here Western blotting experiments confirmed strong regulation of this protein in transgenic but non-tumor bearing mice but in EMSA band shift assays addition of the antibody resulted in a strong band in the pocket of the gel particularly with nuclear extracts of transgenic animals and less so with those of tumor tissue while with non-transgenic controls no band shift could be observed (Figure 15B). Similarly, expression of GR was repressed in nuclear extracts of tumor bearing mice (Figure 15C) while its DNA binding activity was similar at a gene specific promoter sequence of the CAV gene (Figure 15D). Regulation of the p53 protein was also investigated and found to be strongly induced in transgenic but non-tumor bearing mice where as its expression was similar with nuclear extracts of control and tumor bearing mice (Figure 16A). Here, DNA binding activity at gene specific promoters of Xlr, Nr2f1, Zbtb7b, Sprr2i, Ffg18 and ERBB3 was observed with nuclear extracts of transgenic and tumor bearing mice but the overall activity varied considerably (Figure 16b) with no obvious trend. Finally, expression of the hepatic nuclear transcription factors and of COUP-TF was studied. Notably, several of the liver enriched TFs were repressed in nuclear extracts of tumors as were COUP-TFI and II (see Figure 17).

## Discussion

This study aimed at an improved understanding of molecular events associated with EGF-induced, nongenotoxic hepatocarcinogenicity. We report on results of computational as well as experimental analyses carried out on gene expression profiles of successive disease stages, which we described previously [1]. In the course of this work clinical and biological aspects of observed expression changes were examined. Pre-tumor and tumor state expression of 39 known liver cancer biomarkers disclosed possibilities to refine their application as pre-tumor and tumor markers. The marker genes Myc, Glul and Oat presented pre-tumor-specific differential expression which was reverted in the tumor state, whereas Ccnd1, Gpc3, Mvk, Pparg, Rbl2, Robo1 were only upregulated in tumors. The adverse regulation of Pparg combining pre-tumor downregulation with upregulation in tumor invites further investigation as a liver cancer biomarker that supports distinction of three different biological conditions.

### Comparison of GO biological enrichment and network cluster analysis

To further elucidate the biological and molecular functions targeted by expression responses, we analyzed associations between GO biological processes and DE genes in transgenic and tumor cells and constructed network clusters, which connected signaling molecules in pathways with a minimal proportion of differentially expressed components. Since we noticed that genes were often detected in only one progression state by statistical differential expression analysis while fold changes indicated a difference to the normal condition in both states, we constructed so-called extended gene sets to reduce the effect of false negative findings in the statistical analysis step. This was done in a careful manner by taking detection of differential expression in one progression state as evidence for the other and included a gene in the extended set if the detected microarray probe set suggested more than 2-fold expression change. By this process we obtained enlarged up- and downregulated gene sets, which eliminated some differences seen otherwise and led to a more stringent definition of specific up- or downregulation. This was in line with our goals to perform comparative analyses of GO category enrichment and to compare clusters of differentially expressed signaling molecules, where we sought to minimize the possibility of falsely identifying differences.

Comparison of GO biological enrichment pinpointed regulation of genes involved in cell cycle, developmental pathways, lipid metabolism, and protein deubiquitination. Other scientific works supported our findings. In contrast to cell cycle and developmental categories whose enrichment in both transgenic and tumor gene sets reflects progressive regulatory alterations during carcinogenesis, downregulation of genes associated with protein deubiquitination, a relatively new target for cancer therapy, was first observed in tumor cells. Focusing analyses on the identified cellular functions could thus help to further dissect the causal mechanisms of switching from pre-tumor to tumor state. A first step in this direction was undertaken in this work by analyzing and comparing overrepresentation of transcription factor binding sites in promoters of cell cycle as well as lipid metabolism gene sets as further discussed below.

The interplay of differentially expressed signaling molecules was examined in more detail by network analysis. ExPlain generated the network clusters in a data driven process, which is guided by the specified input set of molecules. The resulting clusters represent context-specific subdistricts of the entire cellular network, which are densely populated with molecules targeted by observed expression changes. The network clusters constructed for upregulated transgenic and tumor molecules connected components of growth factor signaling, cell cycle regulation, as well as chemokine and cytokine signaling. We assume that the interplay of molecules with different canonical functions provides for a realistic view on cellular control mechanisms. These are implemented by a cellular network that connects thousands of molecules, many of which exert a regulatory role in a multiplicity of biological contexts. Comparison of transgenic and tumor networks supported our hypothesis of progressive alterations of cell cycle regulation as well as of lipid metabolism during hepatocarcinogenesis. Moreover, this analysis yielded testable hypotheses about relevant molecular cascades involving p107, p130, and p15INK4b as well as survivin, Cdk1, cyclin B1, Plk1, and Bub1. In the transgenic network we found a cascade of molecules regulating cell migration and adhesion, which included VCAM-1, alpha4-integrin, TSP-1, and IAP. This finding complied with decreased enrichment of cell motility components in tumor revealed by GO analysis. Hence, the constructed network clusters complemented the results of our GO analysis by facilitating detailed insight into the molecular pathways targeted by carcinogenic expression changes. Taking into account the presence of EGF-receptors ErbB1-3 in both transgenic and tumor clusters, the networks reveal in addition how components of biological processes proposed by GO analysis were tied to EGF-signaling in the context of hepatocarcinogenesis.

### Reconstruction of regulatory causes of EGF-induced hepatocarcinogenicity

After elaborating on downstream effects of EGF-induced tumor development, we reconstructed causes of observed expression changes. First, we analyzed overrepresentation of transcription factor binding sites in promoters of upregulated and downregulated transgenic as well as tumor gene sets. For this part of the work, we developed a novel statistic for binding site enrichment analysis that compares foreground/background binding site proportions with promoter proportions and quantifies overrepresentation with the ratio quantile of corresponding Beta distributions. The statistic was proposed for several reasons. PWM-based binding site prediction typically requires specification of a score threshold that determines true and false positive rates. In a comparative method, e.g. like F-MATCH, the threshold at which a weight matrix optimally detects overrepresentation is usually not known a-priori. Therefore, F-MATCH adopts the strategy of iterating over score thresholds to optimize the overrepresentation of predicted binding sites for each specified PWM. However, at low score cut-offs, where high numbers of binding sites are predicted in foreground and background promoters, statistical tests like the exact binomial test can report highly significant P-values for small “fold” differences when these are supported by high counts of binding site instances. Consequently, one cannot start from arbitrarily low PWM score thresholds in order to find the best, most likely higher cut-off. Eventually, one might also like to prioritize binding site motifs and would naturally assign highest priority to motifs with strongest enrichment. In this case, simply calculated odds ratios may result in a different ordering of motifs than corresponding enrichment P-values. We therefore developed an approach that focuses on the magnitude of overrepresentation expressed as a probabilistic estimate of the odds ratio of binding sites and promoter sequences in foreground and background gene sets. This quantity cannot be trapped by highly significant P-values associated with small odds ratios, because it focuses on a (statistically corrected) estimate of the odds ratio itself. Finally, we assume that the proposed statistic enables more intuitive prioritization of motifs by expressing their importance as ”relative enrichment” in foreground promoters. It may however by perceived as potential drawback to specify the quantile interval as a free parameter, which was set here to 1%.

In this study, we applied the new promoter analysis method to transgenic and tumor promoter sets and subsequently compared the results of both progression states. Similar to comparison of GO analyses, this setup enabled us to not only identify highly enriched binding sites in promoters of DE genes, but also to observe differences in importance of certain motifs for transgenic and tumor gene sets. As described in the results section, promoter analysis supported stronger regulation of cell cycle and of lipid metabolism in the tumor state by associating motifs of Atf3, Jun, E2f3, and Pparg with corresponding tumor genes and thus supported our previous findings. Importantly, this part of our study revealed overrepresentation of POU motifs predominantly in transgenic promoter sets. Expression profiles of POU factors as well as of HMG and Forkhead factors whose motifs were also identified through promoter analysis led us to propose Oct4 (Pou5f1), Tcf7, Lef1, and Foxc1 as regulators of a transcriptional network potentially under control of Wnt signaling. We speculate that the factors contribute to regulation of developmental pathways, which were enriched in both up- and downregulation according to GO analysis. Although network analyses carried out in this work did not reveal a link between Oct4 and EGF-signaling on the level of protein-protein interactions, in-vivo binding fragments for c-Myc, c-Jun, and c-Fos were previously located in the vicinity of the human POU5F1 gene [25] and provide for a possible explanation for EGF-induced transcriptional activation of Oct4. Altogether, overrepresentation of binding sites in promoters of DE genes detected biologically meaningful transcription regulators, which further support the results of other parts of this study. Subsequent analysis of key nodes upstream of transcription factors predicted for transgenic and tumor promoter sets revealed a switch from EGF signaling in the pre-tumor state to IGF-2 in the tumor state. Indeed, EGF expression was significantly downregulated in tumors and fold changes of other EGF pathway components such as BTC and ErbB3 were decreased in tumors compared to transgenic cells. Furthermore, the overlap of EGF and IGF-2 pathways, as demonstrated by merging both networks into a single pathway, could enable IGF-2 to take over the functional role of EGF and thus render the tumor cell independent of EGF signaling. By the same mechanism cancer cells may acquire resistance to EGF pathway inhibitors.

To further examine the molecular causes of EGF-induced malignant transformation, we searched promoters of upregulated tumor genes for pair wise combinations of TF binding sites. Paired site searches started out from individual site predictions adjusted to a P-value of 10^-4^. We used actual promoter sequences to estimate respective PWM score thresholds corresponding to the chosen background frequency. However, promoter sequences contain both functional and non-functional binding sites. Moreover, the proportion of functional sites among all predicted sites is expected to increase with higher, more stringent PWM scores, so that the background frequency of site predictions could be overestimated by using sequences of regulated genes. Yet, gene expression data allow for selection of sequences in which binding sites should occur at a background rate in the context of the particular biological condition. Thus, we composed background sets of genes with unaltered expression in the tumor state to adjust score cut-offs. In order to focus our analysis on TFs whose activity can be modulated by EGF signaling, we selected motifs of TFs downstream of EGF according to network analysis and of TFs upregulated in the tumor state. As a result, we obtained significantly enriched (P-value <0.001) pairs of TRANSFAC motifs representing several upregulated and EGF-associated TFs. The set of weight matrices selected by co-occurrence analysis was subsequently used as starting point to derive more complex promoter models. Although Fisher test P-values indicated enrichment of several PWM pairs at the chosen significance level, we noticed that each particular combination was present in only few promoters. We attribute this to the stringent threshold that was imposed on individual binding site predictions. Furthermore, we assume that the foreground sets comprised promoters controlled by a number of different regulatory processes, so that higher order TF combinations were more suitable to describe subsets of coregulated promoters.

Based on more complex TF modules, we selected a small set of factors for experimental validation. Results of the EMSA analysis are provided in Table S8. Western blotting and EMSAs demonstrated that c-Myb was upregulated in EGF-transgenic animals and in tumors. Notably, c-Myb is well known for its oncogenic potential and was the subject of targeted therapies in various cancers [26]. We also investigated DNA-binding activity of c-Myb on novel predicted gene targets. These included Bmp7, Pparg and Igf2, all of which play an important role in cancer biology. Indeed, PPAR-gamma antagonists are clinically evaluated for their anti-tumor growth activity in liver cancer. It is of considerable importance, that c-Myb regulates Igf2 (Figure 9), which allows tumor cells to develop an independent autocrine loop thereby integrating EGF and IGF-2 signaling networks [27], [28]. Additionally, IGF proteins are primarily produced in the liver to act as an important pro-survival factor as shown in a number of cancer cell lines.

We identified Stat5 transcription factor binding site (TFBS) enrichment in genes regulated in HCCs. Several studies have demonstrated an important role of STAT5 in liver fibrosis and cancer development through TGF-beta and STAT3 activation. We observed loss of STAT5 protein expression in HCC and demonstrated STAT5 DNA-binding activity in Igfbp6 (Table S8). Indeed, loss of STAT5 causes liver fibrosis and cancer development as recently reported by Hosui et al. [29]. Our computational analyses revealed Igfbp6 to be a target of Stat5 as evidenced by EMSA band shift assays. Recent evidence suggests Igfbp6 to induce cell migration of cancer cells that could be repressed by inhibitors of p38 and ERK1/2 MAPK signaling [30]. Additionally, early studies demonstrated Ets gene regulation in cancer and that overexpression of Ets causes cellular transformation in-vitro as well as in-vivo. The emerging role of ETS in human cancer has recently been reviewed and ETS regulated biological pathways will provide novel opportunities for better diagnosis and staging of disease as well as for the development of anti-cancer therapies [31]. Note, we show Ets-2 DNA-binding for Igfbp6 (Table S8) and therefore identified two TFs (STAT5 and Ets-2) that participate in the regulation of mitogenic IGF proteins.

Furthermore, we detected regulation of genes by C/EBPalpha. Overall we examined 9 novel gene candidates targeted by C/EBPalpha. Strikingly, all TFBS could be confirmed by EMSA band shift assay and in Western blotting experiments with nuclear extracts of tumor liver tissue C/EBPalpha was significantly repressed. This agrees well with its function as a key regulator of p21. Indeed cell cycle progression is regulated, at least in part by protein-protein interaction of C/EBPalpha with p21. It is of no surprise that tumors display less C/EBPalpha expression and we found all novel gene targets of C/EBPalpha to be repressed in tumors (Table S8).

Finally, there is a wealth of information on the role of p53 in liver cancer. Here, we demonstrate p53 binding sites in genes encoding components downstream EGF/IGF-2. Notably, p53 was still identified by promoter analysis although its expression level was similar to normal controls.

In order to summarize the results obtained in this study we have build a hypothetical model-diagram of the EGF/IGF-2 regulatory circuit functioning during the transition from transgenic to tumor state (Figure S2). This model combines two signal transduction networks of the signal flow from EGF and IGF-2 reaching a number of transcription factors (identified by combined promoter and network analysis and validated experimentally), that in turn, regulate expression of several important genes (validated using EMSA assays) that encode components of the upstream network. Thus created feedback loops should play an important role in emerging as well as in stabilizing the cancer state of the cells, In this model, we can propose multiple paths of signals initially coming massively from EGF in the transgenic cells and triggering activity of several TFs, such as C/EBP-alpha, GR and HNF-4alpha, that down-regulate expression of their target gene encoding EGF (Egf) as well as Cav1, thus trying to compensate the excess of the EGF stimulus in the cell. At the same time, through parallel signaling cascades and activation of a number of other TFs, such as c-Ets-1, PPAR-gamma, STAT family factors, c-Myb and others, upregulation of expression of Igf2 gene as well as Igfbp6 and Pparg can be achieved. Due, to several feedback loops on different levels of the network coming from these genes, we can speculate that a steady signal for upregulation of the Igf2 gene leads eventually to a sharp elevation of its expression with the consequence of increase of mitogenic activity of the cells, which marks the transition to the carcinogenic state. It was reported previously, that Igf2 gene is located in an imprinted area of genome and is repressed in most of tissues of the adult organism [32]. Loss of imprinting of the Igf2 gene is one of the most common observations in cancers [33]. It was shown that the imprinting status is maintained by binding of CTCF repressor to an intergenic area of the Igf2 gene and loss of this binding can lead to 10-fold elevation of Igf2 expression [34]. We propose a model where the feedback mechanisms involved in the Igf2 epigenetic control through multiple transcription factors, activators and repressors, play the major role in the switching the cells to malignant transformation.

In conclusion, promoter analysis of the differentially expressed genes enabled us to identify transcription factor binding sites. Such integration of sequence information into signal transduction networks enabled an identification of key nodes upstream of the identified transcription factors. By searching for pairs of TF sites and integration of this information into the network analysis robust information can be retrieved in an unbiased manner that clearly identifies keynodes and molecules acting in concert in defined biological conditions. Therefore, we propose a sequence of events whereby the insulin-like growth factor (IGF) pathway represents an important molecular switch in malignantly transformed liver cells. Possibly, an initial upregulation of EGF is followed by a subsequent and sustained activation of IGF2 signaling cascades. Overall, we hypothesize a switch in autocrine signaling to foster tumor growth that was initially triggered by EGF. In this regard c-Myb is considered to be an important factor of the IGF2 positive feedback loop. Notably, we identified c-Myb binding sites in the promoter IGF2 gene and c-Myb to be a downstream partner of the IGF2 signaling cascade. Therefore our analysis demonstrates the knowledge gain form promoter analysis combined with upstream key node identification.

## Materials and Methods

### Ethics Statement

All animal work followed strictly the Public Health Service (PHS) Policy on Humane Care and Use of Laboratory Animals. Formal approval to carry out animal studies was granted by the ethical review board of Hannover/Lower Saxony, Germany (“Niedersächsische Landesamt für Verbraucherschutz und Lebensmittelsicherheit (LAVES)”, http://www.laves.niedersachsen.de). The approval ID is Az: 33.9-42502-04-06/1204.

### Gene expression profiles of IgEGF-overexpressing murine hepatocytes in transgenic and tumor state

We analyzed gene expression data from murine primary hepatocytes in normal cells as well as different states of disease progression, which were measured on Affymetrix MG_U74Av2 chips. These data were previously described in [1]. For the purpose of studying mechanisms of tumor onset we focused on a subset of hybridizations comprising normal (four replicates), transgenic (three replicates), and small (one replicate from pooled samples) as well as medium-size (four replicates) tumor states. In the transgenic condition hepatocytes overexpressed IgEGF, yet livers presented no detectable tumor, so that respective expression profiles were considered to present a pre-tumor state. The Bioconductor method EBarrays [35], [36] inferred differential expression based on MAS 5 processed expression data, separately comparing transgenic and tumor measurements to the normal condition. Differential expression was assumed for probe sets if the respective posterior probability was greater than 0.5 according to the Lognormal-Normal and the Gamma-Gamma model.

### Analysis of gene expression data with ExPlain

Computational analyses used version 2.3 of the BIOBASE ExPlain system [37], [38]. ExPlain integrates genomic information with biological knowledge bases and computational analysis methods. As described below, transcription factor binding sites (TFBSs) were predicted by positional weight matrices (PWMs) from the TRANSFAC database [39] in murine promoters specified in the TRANSPro database [40]. The latter resource provided 61,113 transcription start sites (TSSs) and surrounding genomic sequences for 24,353 murine genes. TRANSPro defines a set of reference TSSs for each gene by a weighted combination of annotations from EPD, DBTSS, Ensembl, and Fantom [41]–[44]. Information from manually curated databases is given higher weight than computationally predicted TSS locations. A score is assigned to each reference TSS according to spatial density and weights of relevant primary TSS annotations and the reference TSS with the highest score among several alternative TSSs of a particular gene is denoted “best supported”. Furthermore, topological analyses of signal transduction networks were performed using molecular reactions collected in the TRANSPATH database [45], covering more than 130,000 reactions and interactions between about 27,000 genes and 86,000 proteins (enzymes, transcription factors, receptors, adaptor proteins), their complexes, modified forms, small molecular ligands, and endogenous metabolites.

### Functional analysis of differentially expressed genes

The Gene Ontology (GO) [46] provides an extensive ontological description of cellular components, molecular functions and biological processes. It is routinely applied in studies to test for enrichment of categories in sets of genes or proteins. Statistical significance of enrichment is typically quantified by the one-tailed Fisher test. The one-tailed Fisher test calculates the probability *P(X ≥ x)* of finding purely by chance at least *x* out of *K* genes associated with a category comprising *M* genes given a total database size of *N* genes (1).
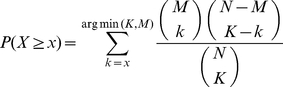
(1)


To gain insight into functional changes accompanying tumor development in non-genotoxic hepatocarcinogenesis we compared enrichment P-values of GO biological process terms calculated for differentially expressed genes of transgenic and tumor states. Fisher test P-values were calculated for all GO terms linked to at least one gene in either gene set or otherwise assigned a value of 1.0. Functional categories were then attributed to the gene set (transgenic or tumor) achieving the highest significance of enrichment and ranked by the difference of log_10_-P-values in order to guide towards cellular functions most strongly affected by disease progression.

### Identification of key nodes and clusters in signal transduction network using a graph-topological algorithm

Signal transduction through a network of molecules is an important part of the cellular regulatory system. We applied network analysis methods to elucidate the molecular context of differentially expressed signaling molecules (network clusters) and to reconstruct pathways between transcription factors and upstream regulators of their activity (key nodes). Network construction in ExPlain implements the Dijkstra algorithm as core to find the shortest path (minimal cost) tree whose reaction cascades are weighted by the sum of the involved edge costs. We initialized the edge costs using three different values. Cost 0 was used to represent hierarchical classification links between molecules in the database. For direct reactions and indirect reactions we used cost 1 and 3, resp. A path cost is implicitly defined as the sum of the costs of its edges.

The network cluster algorithm constructs cascades of maximally three reactions to connect each pair of input vertices (molecules). As a result, input molecules are joined into clusters of one or more proteins linked by corresponding pathways.

In key node analysis, we searched for signaling molecules (key nodes) and corresponding networks that can transmit a signal to or receive a signal from several of input molecules within a certain path cost limit [41]. A key node search starts from each molecule of an input set *V_x_* and constructs the shortest path to all nodes of the complete network *V* within a given maximal path cost *d_max_* (i.e, the sum of the costs of all edges in the shortest path from a vertex in *V_x_* to a vertex in *V* should be smaller than or equal to *d_max_*). The search can be conducted in reverse direction of the edges leading to input molecules (upstream) or in the same direction (downstream). For each node *i* of *V*, the algorithm counts *N_i_*, the number of nodes of *V_x_* (number of true positives) that can be reached by a path satisfying *d_max_*. The list of all possible key nodes that can reach at least one input molecule is sorted according to the specificity score *s_i_*.
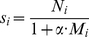
(2)


In equation (2), *M_i_* is the number of molecules in the whole network, which are reachable from node *i* within *d_max_* steps, yet are not part of the input set of molecules (false positives). The parameter *α* is a penalty *(0<α<1)* that adjusts the balance between true positives and false positives. As described earlier [41], we empowered the key node analysis by the so-called “pathway persistence”. This extension integrates information about known canonical pathways and chains of consecutive reactions that were proven experimentally in order to improve the accuracy of key node prediction, especially to diminish the false positive error. Briefly, the modified algorithm gives preference to inclusion of verified reaction cascades (canonical pathways) into shortest paths by on-the-fly insertion of additional edges, which represent short-cuts within verified reaction cascades. The desired effect is that the key node algorithm not only prefers reactions from the verified cascades, but at the same time, it is pushed to stay (“to persist”) within one cascade as much as possible. The strength of this effect is adjusted by a parameter *h (h [0,1])*.

In details, the application of verified cascades (pathways) to the key node search is done as follows: A pathway *P* is defined as a graph *G_P_  = * (*V_P_, E_P_, C*), which is a sub-graph of the complete signaling graph *G =  (V, E, C)*. Let *S_Pij_* be the graph of the shortest paths between *i,j* *V_P_* within pathway *P*. Further, let *C_Pij_* be the cost of the shortest paths, where *C_Pij_*  =  ∞ if *S_Pij_*  =  Ø. We combine *G_P_* and *G* yielding the final graph *G'* = (*V*,*E'*,*C'*) by introducing additional edges *E'  =  E U {(i, j) | S_Pij_ ≠ Ø}* and by extending the cost function for them by *C'  =  {f(C_Pij_)| f(C_Pij_) ≤ C_ij_} U {C_ij_| f(C_Pij_) > C_ij_}*. As function *f* we use *f*(*x*)  =  *x^h^*, with *h [0,1]*. The aim of *f* is to make the cost function of any new edge *(i, j)* sublinearly dependent from the cost of the corresponding original shortest path *S_Pij_*. The effect is that the costs of paths within a known pathway have decreased due to the cheaper short-cuts. This algorithm prioritizes the selection of the paths persisting inside known pathways during the search of the key nodes. The effect is maximal when *h* = *0* and absent when *h* = *1*. Therefore we call *h* “rigidity parameter” which the balance between rigidity and sensitivity during the search. As demonstrated earlier, the pathway persistence has an advantageous effect with regard to sensitivity and specificity of identifying correct pathway components in a key node network [41].

Due to the strong connectivity in signaling networks, a key node search is prone to yield many false-positive key nodes. In this study, we therefore calculated an empirical cumulative probability (ECP value) for each key node identified, based on the score rank of the key node. The ECP value of a key node is calculated as ECP = RK*1/NK, where, RK is the rank of the key node and NK is the total number of the key nodes found in the analysis. ECP estimates a cumulative probability of a given key node to be found at the rank RK and higher by random chance. We then contrasted ECP values of key nodes obtained for the transgenic state with ECP values of key nodes of the tumor state. This was done to highlight those key nodes with strongest differences regarding their importance for transgenic and tumor TFs as well as expression changes and thereby rely less on selection of key nodes based on statistical error rates. A framework to automatize handling of the various free parameters required by the key node analysis algorithm and to estimate false discovery rate using random shuffling of the input nodes shall be described elsewhere (manuscript in preparation).

### Promoter analysis

#### Overrepresentation of TF binding sites

We analyzed overrepresentation of transcription factor binding sites in promoters of differentially expressed genes and compared the importance of transcription factors for transgenic and tumor gene sets. It is well known that promoters of co-regulated genes are enriched with binding sites of relevant transcription factors [47]. Several algorithms have been developed that test for the significance of enrichment of binding sites in promoters of a gene set of interest (foreground set) compared to a background, where the background set is often compiled from genes which are not differentially expressed in the same biological condition or from randomly sampled genes. For this type of analysis, ExPlain provides the F-MATCH program described in [41]. The algorithm starts with PWM score thresholds employed in an initial site search conducted with MATCH [48] and iteratively increases the threshold of each individual PWM to find a parameter that produces the most significant enrichment of sites in foreground promoters. Significance of enrichment is quantified by the binomial exact test calculated according to equation (3).
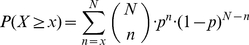
(3)Here *n* denotes the number of predicted sites in the foreground and *N* is the total number of sites. The relative proportion of foreground sequences among both foreground and background sequences gives the parameter *p*. Hence, the F-MATCH algorithm compares the proportion of foreground sites among all predicted binding sites to the proportion of foreground promoters among all promoters.

We adopted this strategy to juxtapose the relative importance of transcription factors (represented by their PWM) for corresponding transgenic and tumor gene sets. Instead of using P-values to quantify enrichment of binding sites in a foreground promoter set, we expressed the relative importance of a TF by a probabilistic estimate of the odds ratio of site and promoter proportions. As in the F-MATCH algorithm, PWM score thresholds were optimized to yield a maximal odds ratio. Given counts of predicted binding sites as well as counts of promoters, we assumed independent Beta distributions (4) for the foreground proportions.
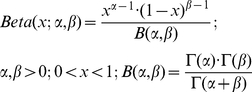
(4)Regularizing observations with a uniform prior, *Beta(α = 1,β = 1)*, promoters were assumed to come from the distribution *Beta_p_(α_p_ = p_f_+1,β_p_ = p_b_+1)*, where *p_f_* and *p_b_* denote the number of promoters in foreground and background, respectively. Likewise, the site distribution (at a certain PWM score threshold) was set to *Beta_s_(α_s_ = n_f_+1,β_s_ = n_b_+1)*, with *n_f_* and *n_b_* denoting counts of predicted binding sites in foreground and background. These two Beta distributions were used to assess the uncertainty about true proportions of sites as well as promoters.

For two independent distributions the probability *P(w = x_s_/x_p_)*, with *x_p_ ∼ Beta_p_* and *x_s_ ∼ Beta_s_*, can be computed from the joint density. An exact expression for *P(w = x/y)* was derived by Pham-Gia [49] and is recapitulated in (5).
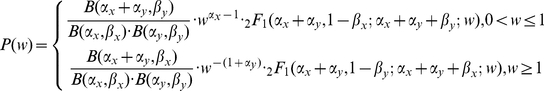
(5)


To avoid numerical difficulties with evaluating the Gauss hypergeometric function *_2_F_1_* near *w = 1*, we calculated *P(w)* by numerical integration of equation (6).
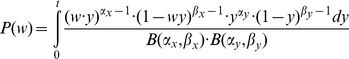
(6)where
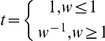
Equation 6 can be evaluated in log-space before exponentiation, which can be beneficial for large *α'*s and *β'*s. Eventually, we used equation (6) to compute quantile (F^−1^) estimates of the ratio of site and promoter proportions by numerical integration. The p-quantile yields the value for which the cumulative probability is at most p. In this work, we chose p = 0.01, so that the ratio of corresponding Beta distributions was assumed to be at least as high with 99% probability. The computer program sought a PWM score threshold to maximize this quantile value. Hence, a value F^−1^(0.01) = 2 for a certain PWM means that the proportion of foreground sites among all predicted sites (foreground + background) was at least two times higher than the proportion of foreground promoters among all promoters with 99% probability which indicates enrichment of sites in foreground promoters.

### Calculation of P-values for MATCH scores

PWM score thresholds for initial MATCH searches were adjusted to a common background frequency as baseline. Single binding site analyses used initial theoretically calculated cut-offs corresponding to a P-value of 0.05. P-values for PWM scores were computed with the standard method [50], which was extended to consider searches over both sequence orientations and to adopt a dinucleotide model for random sequences as described in the following.

Let *Q  =  (q_ik_)* be the *W x 4* score matrix with *i  =  1. .W* vectors, in following called site positions, each with *k  =  1. .4* scores for residues *R  =  (r_k_)  =  (A,C,G,T)*. This matrix is used to score an alignment of the TFBS profile with a sequence segment of length *W*. The score function is additive, so that the score *S* of a sequence segment is evaluated by summing up residue scores of all site positions. Further, let *π  =  (π_k_)* be a mononucleotide background model. Usually, one considers both orientations of the sequence or equivalently both PWM orientations. A method for P-value calculation should take this into account and determine the probability that either score (forward or reverse orientation) satisfies a given threshold. The reverse PWM *Q'* is defined in (7) and (8).
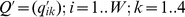
(7)

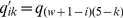
(8)Given forward and reverse orientations, there is a pair of scores *(q_ik_,q'_ik_)* for each position of an alignment and subsequently a pair of scores *(s,s')* for the sequence segment. As previously shown, the probability distribution of PWM scores can be efficiently calculated by convolution [50]. The latter can be extended to calculate the joint probability density *f(s,s')* by the convolution defined in (9). Equation 9 gives the joint probability density of scores *s_i_* and *s'_i_* up to site position *i* by convolution of *g(q_ik_,q'_ik_)* and *h(s_i_-q_ik_,s'_i_-q'_ik_)*, where *g* is the function of score pairs at site position *i* of *Q* and *Q'* and *h* is the joint probability density of score pairs up to position *i-1*.
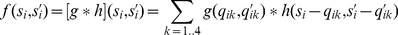
(9)When a mononucleotide background model is used to calculate the false positive rates of PWM scores, *g(q_ik_,q'_ik_)* is just the mapping *g: q_ik_,q'_ik_ → π_k_*. The cumulative probability 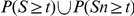 that determines selection of score threshold *t* is derived from the joint probability density of scores.

We further extended the standard method to apply a dinucleotide background model. By conditioning score functions on the terminal residue j associated with a score pair *(s_i_,s'_i_)*, the convolution also accommodates higher-order background models (10).
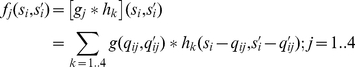
(10)In this study, we employed a dinucleotide model that was estimated from the first 1000 residues upstream of all murine TSSs in TRANSPro. We selected this particular region for several reasons. First, start positions of 74.4% of the genomic TFBS entries in TRANSFAC 12.1 map to this sequence range. The distribution of TSS-relative locations of known binding sites is shown in Figure 1. While it is well known that transcription factors can exert an effect on promoters that are located thousands of base pairs away, TRANSFAC data suggest that functional binding sites are predominantly located in the proximal upstream region of the TSS. The distribution shown in Figure 1 has a peak at position –115 (red line). Also, locations of binding sites identified by ChIP-chip or ChIP-seq are often overrepresented in the TSS vicinity (data not shown). Second, we did not include downstream residues to avoid inclusion of coding regions.

### Co-occupancy of TF binding sites

An analysis of individual TFBS with a test for pair wise co-occurrences of binding sites was performed. The approach represents a variant of the F-MATCH algorithm for binding site pairs and quantifies overrepresentation of promoter sequences with sites of both PWMs in the foreground set using the Fisher test. Like F-MATCH, the site co-occurrence method starts with externally defined score thresholds that can be further optimized using minimization of the Fisher test P-value as objective. While sequences containing a certain binding site pair can be significantly overrepresented in the foreground set, the association of the two sites might not be significant per se, e.g. enrichment may be determined by only one type of sites. Therefore, the signal conveyed by the event of co-occurrence itself was controlled using a stringent site score cut-off (P-value 10^-4^). Furthermore, the frequency of sequences with both site types was compared with the expected frequency in the foreground, given the numbers of promoters with either TFBS, and calculated a coefficient of independence (11), which is similar to the mutual information of two random variables.
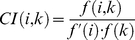
(11)In (11), *f(i,k)*, *f(i)* and *f(k)* denote proportions of promoters with the TFBS pair, site *i*, and site *k*, respectively. To further consider a pair, it was required for this value to be at least 1.3. Finally, dependence of PWMs with similar sequence specificity was eliminated, because this property would inevitably produce high co-occurrence rates. This problem was addressed by considering only pairs of PWMs showing overlap in less than 10% of predicted sites. This approach was fully data-driven since admissible PWM combinations, baseline thresholds, and final score cut-offs, for individual PWMs and for the pair, were estimated on the basis of a background promoter set that was defined by the gene expression data.

## Supporting Information

Table S1 Differentially expressed genes detected by Ebarrays in transgenic and tumor cells.
(XLS)

Table S2 Biological processes selected by comparison of their enrichment P-values in transgenic and tumor state.
(XLS)

Table S3 Upregulated molecules connected in network clusters shown in Figures 4 and 5.
(XLS)

Table S4 TRANSFAC weight matrices with highest q-values in transgenic and tumor gene sets.
(XLS)

Table S5 Transcription factors represented by enriched TRANSFAC motifs.
(XLS)

Table S6 TRANSFAC PWM pairs enriched in upregulated tumor genes (P < 0.001).
(XLS)

Table S7 Transcription factors identified by PWM co-occurrence analysis.
(XLS)

Table S8 Experimental validation of predicted binding sites in promoters of up- and downregulated genes.
(XLS)

Figure S1 Multiple alignment of TRANSPro promoters (-1000 to +500) of murine Bcl2a1a-d.
(TIF)

Figure S2 Representation of EGF/IGF-2 regulatory circuit in SBGN notation. This diagram was constructed using the geneXplain platform for systems biology (www.genexplain.com) and adapted with the Inkscape SVG editor (inkscape.org). The SBGN diagram illustrates the feedback loops triggered by EGF and IGF-2 signaling. The endpoints of regulation – multiple transcription factors (shown in light blue) that are activated through upstream signaling events, regulate expression of their target genes (shown in light blue) whose products are the key components of the signaling network (shown in red) upstream of the transcription factors.
(PNG)
