# Supplementary material for: Advanced Computational Biology Methods Identify Molecular Switches for Malignancy in an EGF Mouse Model of Liver Cancer
Source: PLoS One. 2011 Mar 28;6(3):e17738. doi: 10.1371/journal.pone.0017738 (PMC3065454; doi:10.1371/journal.pone.0017738)
Supplement: Table S2 — Biological processes selected by comparison of their enrichment P-values in transgenic and tumor state. (XLS) [file pone.0017738.s002.doc]

Bold-typed gene symbols were specifically differentially expressed in the respective cell state (sets 1, 5, 6, and 10 of Table 1).

| GO Biological process | **Transgenic** |  |  | **Tumor** |  |  |
| --- | --- | --- | --- | --- | --- | --- |
|  | **Gene list** | **# Genes** | P-value | **Gene list** | **# Genes** | **P-value** |
| Upregulation |  |  |  |  |  |  |
| Cell division | Anln, Birc5, Bub1, Ccnb1, Ccne2, Cd2ap, Cdc20, Cdc23, Cdc2a, Hells, Kif11, Kif20a, Kif23, Ncaph, Nedd9, Nusap1, Prc1, Rock2, Sept14, Sept6, Smc4, Top2a | 22 | 5.73E-08 | Anln, **Aurkb**, Birc5, Bub1, Ccnb1, **Ccnb2**, **Ccnd1**, Ccne2, Cd2ap, Cdc20, Cdc23, Cdc2a, Hells, Kif11, Kif20a, Kif23, **Mcm5**, Ncaph, **Ndc80**, Nedd9, Nusap1, Prc1, **Racgap1**, Rock2, Sept14, Sept6, Smc4, Top2a, **Wee1** | 29 | 2.29E-12 |
| Cell cycle | Anln, Anxa1, Aurka, Birc5, Btc, Btg3, Bub1, Ccdc47, Ccnb1, Ccne2, Cd2ap, Cdc20, Cdc23, Cdc2a, Cdkn3, Ckap2, E2f3, Ern2, **Foxc1**, **Gadd45a**, Hcfc1, Hells, **Hic1**, **Hus1**, Kif11, Kif23, Ltb, Mki67, **Myc**, Ncaph, Nedd9, Nusap1, Plk1, Prc1, Rnf2, Sept14, Sept6, Shc1, Smc4, Stmn1, **Uhmk1** | 41 | 5.42E-07 | Anln, Anxa1, **App**, Aurka, **Aurkb**, Birc5, **Bmp7**, Btc, Btg3, Bub1, Ccdc47, Ccnb1, **Ccnb2**, **Ccnd1**, Ccne2, Cd2ap, Cdc20, Cdc23, Cdc2a, **Cdkn2b**, Cdkn3, Ckap2, **Dab2ip**, E2f3, Ern2, Hcfc1, Hells, Kif11, **Kif22**, Kif23, Ltb, Mki67, Ncaph, **Ndc80**, Nedd9, Nusap1, Plk1, Prc1, **Ptprv**, **Racgap1**, **Rad51l1**, **Rbl1**, **Rbl2**, Rnf2, Sept14, Sept6, Shc1, Smc4, Stmn1, **Uhrf1**, **Wee1** | 51 | 9.86E-11 |
| M phase | Anln, Aurka, Birc5, Btc, Bub1, Ccnb1, Cd2ap, Cdc20, Cdc23, Cdc2a, Hells, **Hus1**, Kif11, Kif23, Ltb, Mki67, Ncaph, Nedd9, Nusap1, Plk1, Shc1, Smc4 | 22 | 6.56E-06 | Anln, Aurka, **Aurkb**, Birc5, Btc, Bub1, Ccnb1, **Ccnb2**, Cd2ap, Cdc20, Cdc23, Cdc2a, Hells, Kif11, **Kif22**, Kif23, Ltb, Mki67, Ncaph, **Ndc80**, Nedd9, Nusap1, Plk1, **Ptprv**, **Rad51l1**, Shc1, Smc4, **Wee1** | 28 | 8.24E-09 |
| Mitosis | Anln, Aurka, Birc5, Btc, Bub1, Ccnb1, Cd2ap, Cdc20, Cdc23, Cdc2a, Hells, **Hus1**, Kif11, Kif23, Ltb, Ncaph, Nedd9, Nusap1, Plk1, Shc1, Smc4 | 21 | 1.75E-07 | Anln, Aurka, **Aurkb**, Birc5, Btc, Bub1, Ccnb1, **Ccnb2**, Cd2ap, Cdc20, Cdc23, Cdc2a, Hells, Kif11, **Kif22**, Kif23, Ltb, Ncaph, **Ndc80**, Nedd9, Nusap1, Plk1, **Ptprv**, Shc1, Smc4, **Wee1** | 26 | 2.27E-10 |
| M phase of mitotic cell cycle | Anln, Aurka, Birc5, Btc, Bub1, Ccnb1, Cd2ap, Cdc20, Cdc23, Cdc2a, Hells, **Hus1**, Kif11, Kif23, Ltb, Ncaph, Nedd9, Nusap1, Plk1, Shc1, Smc4 | 21 | 2.52E-07 | Anln, Aurka, **Aurkb**, Birc5, Btc, Bub1, Ccnb1, **Ccnb2**, Cd2ap, Cdc20, Cdc23, Cdc2a, Hells, Kif11, **Kif22**, Kif23, Ltb, Ncaph, **Ndc80**, Nedd9, Nusap1, Plk1, **Ptprv**, Shc1, Smc4, **Wee1** | 26 | 3.64E-10 |
| Downregulation |  |  |  |  |  |  |
| **Lipid metabolic process** | Acaa1a, Acaa1b, Acot2, BC048644, Cav3, Cyp4a10, Cyp4a14, Hmgcs2, **Hsd17b14**, Rdh16, **St8sia5**, **Tsta3** | 12 | 0.0004 | Acaa1a, Acaa1b, Acot2, **Apoa5**, BC048644, **C1qtnf3**, Cav3, **Chpt1**, **Cln3**, **Cryl1**, **Cyp17a1**, Cyp4a10, Cyp4a14, **Cyp7b1**, **Elovl3**, **Fabp7**, Hmgcs2, **Mup1**, **Mup2**, **Mup3**, **Mup4**, **Mup5**, **Nr0b1**, **OTTMUSG00000007485**, **Pck1**, Rdh16, **Thrsp** | 27 | 1.33E-10 |
