# Supplementary material for: Advanced Computational Biology Methods Identify Molecular Switches for Malignancy in an EGF Mouse Model of Liver Cancer
Source: PLoS One. 2011 Mar 28;6(3):e17738. doi: 10.1371/journal.pone.0017738 (PMC3065454; doi:10.1371/journal.pone.0017738)
Supplement: Table S3 — Upregulated molecules connected in network clusters shown in Figures 4 and 5. (XLS) [file pone.0017738.s003.doc]

Entries in the Network column indicate whether a molecule was only part of the transgenic network (Tr) or of the tumor network (Tu). Bold numbers: statistically significant differential expression (see Supplement 1); FC: fold change

| Gene symbol | **Molecule name** | **Transgenic FC** | **Tumor FC** | Network |
| --- | --- | --- | --- | --- |
| Adam10 | ADAM10 | **4.12** | **2.63** |  |
| Adora1 | A1R | 1.34 | **2.80** | Tu |
| Aebp1 | AEBP1 | **4.14** | **8.33** |  |
| App | APP695 | 1.57 | **3.30** | Tu |
| Aurka | Aurora-A | 2.27 | **4.48** |  |
| Bcl2l1 | Bcl-xL | **5.61** | **4.12** |  |
| Bcl2l11 | BimEL | **7.04** | **3.95** |  |
| Birc5 | survivin | **3.63** | **8.96** |  |
| Bmp7 | BMP7 | 0.92 | **4.64** | Tu |
| Btc | BTC | **7.19** | **4.92** |  |
| Bub1 | Bub1 | **4.04** | **8.17** |  |
| Ccnb1 | cyclin B1 | 2.73 | **5.38** |  |
| Ccnd1 | cyclin D1 | 1.19 | **2.87** | Tu |
| Cd44 | CD44 | 2.30 | **4.15** |  |
| Cd47 | IAP | **2.85** | 1.36 | Tr |
| Cdc20 | Cdc20 | 2.76 | **4.05** |  |
| Cdc2a | Cdk1 | **5.56** | **14.89** |  |
| Cdkn2b | p15INK4b | 1.76 | **3.53** | Tu |
| Crkl | CrkL | **3.04** | 1.67 | Tr |
| Cxcr4 | CXCR4 | **3.73** | **5.24** |  |
| Dusp6 | MKP-3 | **4.52** | 2.35 |  |
| Dusp8 | M3/6 | **3.54** | **4.92** |  |
| Egr1 | Egr-1 | **10.90** | **7.14** |  |
| Erbb3 | ErbB3 | **6.99** | 2.35 |  |
| Ets2 | c-Ets-2 | **3.94** | **3.57** |  |
| Fgfr1 | FGFR-1 | **3.19** | 3.72 |  |
| Fos | c-Fos | **3.31** | **3.68** |  |
| Hspb1 | Hsp27 | **3.30** | 1.40 | Tr |
| Igf2 | IGF-2 | 2.90 | **25.98** |  |
| Il1rn | IL-1RA | 2.75 | **8.49** |  |
| Il1rap | IL-1RAcP | **3.60** | 1.85 | Tr |
| Itga4 | alpha4-integrin | **4.29** | 0.30 | Tr |
| Jun | c-Jun | **3.17** | **3.18** |  |
| Map3k2 | MEKK2 | **6.82** | 2.17 |  |
| Myc | c-Myc | **6.26** | 1.39 | Tr |
| Nedd9 | HEF1 | **8.14** | **5.38** |  |
| Pfkfb2 | PFKFB-2 | **6.58** | 3.09 |  |
| Pld1 | PLD1 | **4.01** | **3.61** |  |
| Plk1 | Plk1 | **3.04** | **6.41** |  |
| Pparg | PPAR-gamma1 | 0.40 | **5.76** | Tu |
| Rbl1 | p107 | 1.58 | **2.63** | Tu |
| Rbl2 | p130 | 1.11 | **7.27** | Tu |
| Rgs16 | RGS16 | 2.26 | **3.11** |  |
| Rock2 | ROCK-II | 2.66 | **5.87** |  |
| Smad3 | Smad3 | **3.69** | **3.81** |  |
| Thbs1 | TSP-1 | **16.74** | **12.04** |  |
| Vcam1 | VCAM-1 | **3.33** | 2.17 | Tr |
| Wee1 | Wee1 | 1.63 | **3.71** | Tu |
