# Supplementary figures and images for: Advanced Computational Biology Methods Identify Molecular Switches for Malignancy in an EGF Mouse Model of Liver Cancer
Source: PLoS One. 2011 Mar 28;6(3):e17738. doi: 10.1371/journal.pone.0017738 (PMC3065454; doi:10.1371/journal.pone.0017738)

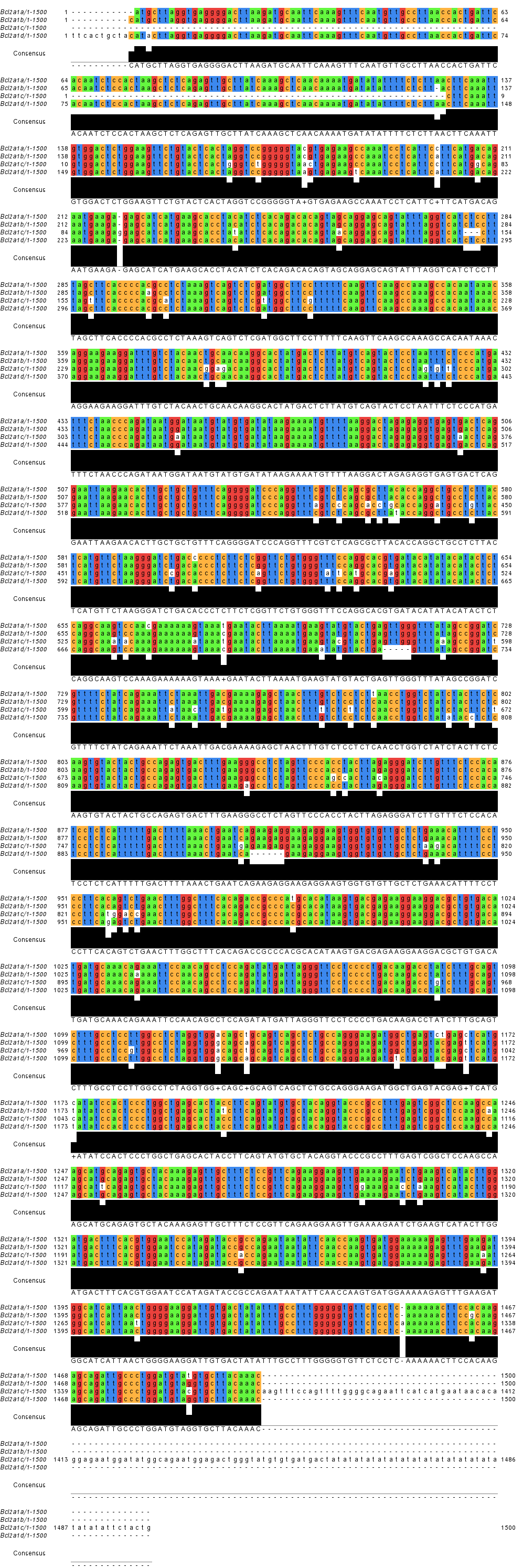

Supplement: Figure S1 — Multiple alignment of TRANSPro promoters (-1000 to +500) of murine Bcl2a1a-d. (TIF) [file pone.0017738.s009.tif]

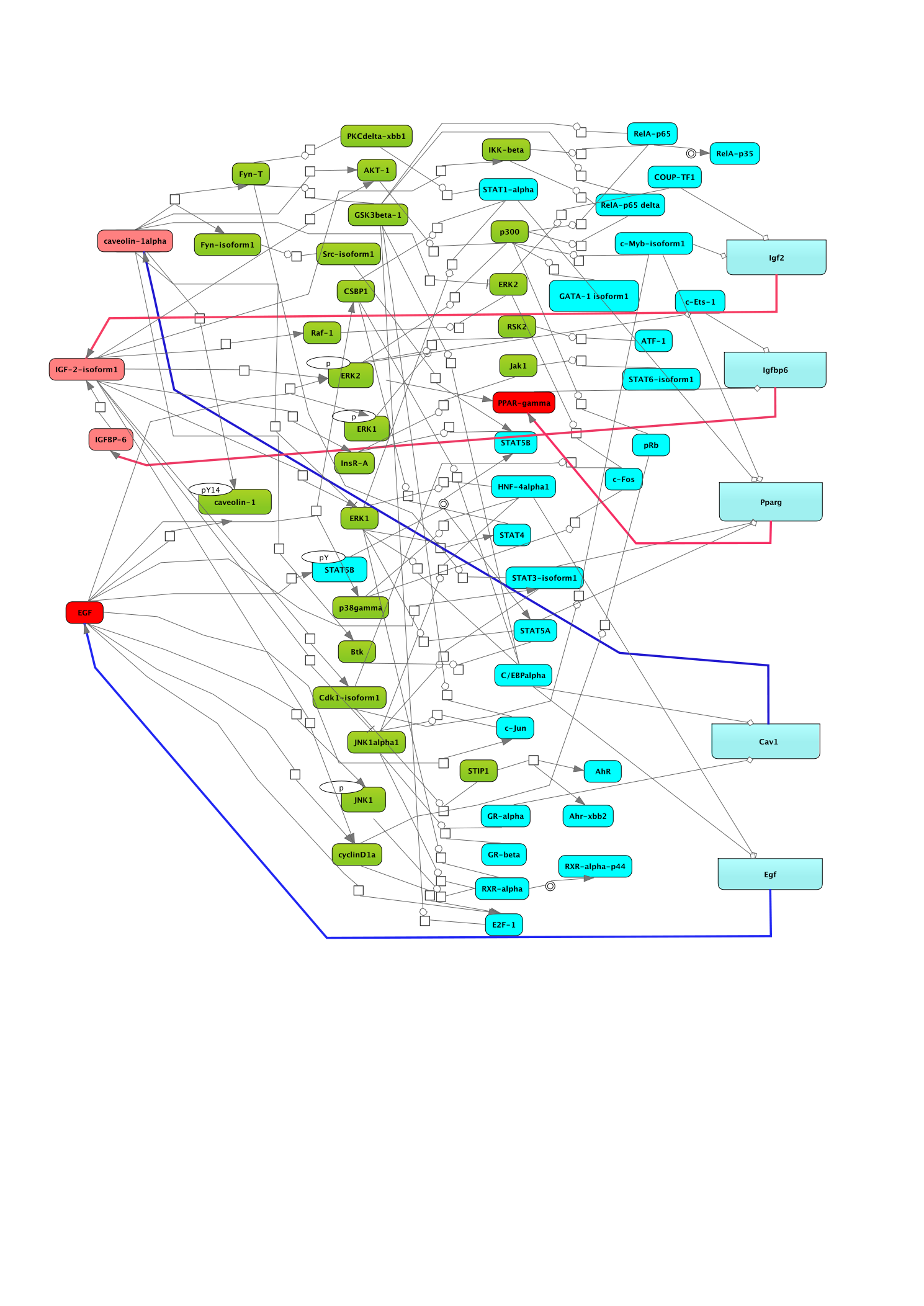

Supplement: Figure S2 — Representation of EGF/IGF-2 regulatory circuit in SBGN notation. This diagram was constructed using the geneXplain platform for systems biology (www.genexplain.com) and adapted with the Inkscape SVG editor (inkscape.org). The SBGN diagram illustrates the feedback loops triggered by EGF and IGF-2 signaling. The endpoints of regulation – multiple transcription factors (shown in light blue) that are activated through upstream signaling events, regulate expression of their target genes (shown in light blue) whose products are the key components of the signaling network (shown in red) upstream of the transcription factors. (PNG) [file pone.0017738.s010.png]
